# The Acute Respiratory Distress Syndrome (ARDS): epidemiology, etiology, molecular mechanisms, diagnosis and therapeutic strategies

**DOI:** 10.1186/s43556-026-00532-2

**Published:** 2026-08-07

**Authors:** Defei Tan, Junlan Zhou, Lingling Li, Yanan Zhong, Ziping Cheng, Tao Yang

**Affiliations:** 1 Department of Pharmacy, Afﬁliated People’s Hospital of Jiangsu University, 8 Dianli Road, Zhenjiang, 212001 China; 2 Department of Respiratory and Critical Care Medicine, Afﬁliated People’s Hospital of Jiangsu University, 8 Dianli Road, Zhenjiang, 212001 China; 3 Department of Respiratory and Critical Care Medicine, Shanghai Tenth People’s Hospital, Tongji University School of Medicine, 301 Middle Yanchang Road, Shanghai, 200072 China

**Keywords:** ARDS, Etiology, Molecular mechanisms, Heterogeneity, Diagnosis, Clinical management

## Abstract

Acute respiratory distress syndrome (ARDS) is a severe clinical condition characterized by acute hypoxemic respiratory failure (AHRF) and diffuse pulmonary inflammation, which can be triggered by diverse intrapulmonary, extrapulmonary, and host-related factors. Over recent decades, basic research and clinical trials have significantly advanced our understanding of ARDS mechanisms, and effective comprehensive critical care interventions, including lung-protective ventilation, prone positioning, and individualized fluid management, have been widely implemented. Nevertheless, ARDS continues to be associated with substantial morbidity and mortality, and its marked clinical and biological heterogeneity remains a major obstacle to effective management and therapeutic translation. This review summarizes current evidence on ARDS epidemiology, etiology, pathophysiology, diagnosis, and clinical management. We also trace the evolution of diagnostic criteria, including the latest global definition, and critically examine disease heterogeneity, recent advances in critical care strategies, emerging therapeutic approaches, barriers to clinical translation, and future research priorities. Particular emphasis is placed on integrating clinical, physiological, and multi-omics data to support biologically and clinically meaningful patient phenotyping grounded in disease mechanisms. Building on this phenotypic stratification, innovative trial designs, including predictive enrichment and adaptive platform trials, may facilitate the development of more individualized, targeted, and dynamically adjusted treatment strategies for ARDS. In summary, establishing a precision medicine framework for ARDS that integrates etiology, mechanistic insights, phenotype-based patient stratification, individualized therapeutic strategies, and long-term management will be essential to address current challenges and improve patient outcomes.

## Introduction

Acute respiratory distress syndrome (ARDS) is a life-threatening clinical condition characterized by acute hypoxemic respiratory failure (AHRF) and diffuse pulmonary inflammation that occurs independently of cardiogenic pulmonary edema [1–3]. It can be triggered by a wide range of direct pulmonary injuries or indirect extrapulmonary [1, 4, 5] insults. Despite the implementation of effective comprehensive critical care interventions, including lung-protective ventilation, prone positioning, and individualized fluid management [6–12], ARDS remains a major global health challenge with persistently high morbidity and mortality.

Currently, no pharmacological therapies are routinely effective in ARDS, and precision-targeted treatments tailored to individual patient characteristics remain elusive. This challenge stems partly from the marked clinical and biological heterogeneity of the syndrome; in addition, potentially reversible causes, particularly the causative pathogens, are often difficult to identify in a timely manner [2], which hinders early initiation of etiology-directed therapy. The molecular and cellular mechanisms underlying ARDS are complex and dynamically evolving, shaped by multiple factors including etiology, disease stage, host status, and immune compartmentalization.

More importantly, clinically relevant biomarkers, genetic features, or phenotypic characteristics that are identifiable, measurable, and targetable remain lacking. As a result, most interventions continue to rely on average treatment effects rather than enabling precise selection of patients most likely to benefit. Meanwhile, conventional clinical trial designs are limited by rigid enrollment criteria, suboptimal timing of intervention, and insensitive endpoint selection, further reducing the ability to detect potentially meaningful therapeutic signals. Accordingly, meaningful advances in ARDS treatment will depend not only on the development of new drugs, but also on the coordinated progress in rapid etiological identification, mechanism-guided stratification, and innovative trial design.

This review synthesizes the latest advances in ARDS research, encompassing its epidemiology, etiology, host susceptibility factors, and pathophysiological mechanisms. We also trace the evolution of ARDS diagnostic criteria and discuss the newly established global definition. Furthermore, we highlight emerging treatment strategies and future research directions, with a specific focus on leveraging clinical, physiological, and multi-omics data, such as single-cell genomics, spatial transcriptomics, and proteomics, for ARDS patient phenotyping grounded in an in-depth understanding of disease pathophysiology. Building upon this phenotypic stratification, the adoption of innovative clinical trial designs, such as predictive enrichment trials and adaptive platform trials, aims to ultimately realize individualized, targeted, and dynamically adjustable treatment regimens for ARDS, thereby effectively improving patient outcomes. Finally, we advocate for moving beyond the traditional paradigm of defining ARDS success solely by discharge outcomes and instead emphasize long-term, multidimensional support for survivors and their families. In summary, establishing a precision medicine framework for ARDS, anchored in etiology, mechanisms, phenotypic diagnosis, treatment, and prognosis, and focused on treatable traits, will be a pivotal yet challenging endeavor in the field over the next decade.

## Epidemiology

The incidence and mortality of ARDS vary substantially across global regions. Reported incidence ranges from 3.65 to 78.9 cases per 100,000 person-years [2, 13]. The Large Observational Study to Understand the Global Impact of Severe Acute Respiratory Failure (LUNG-SAFE) [14], which applied the Berlin definition [15], found that ARDS affected 10.4% of intensive care unit (ICU) patients. In contrast, studies from mainland China reported a lower incidence of ARDS but higher mortality and rates of withdrawal of life-sustaining care among patients with moderate-to-severe disease [16].

Demographic factors, including age, sex, race, and ethnicity, influence both the incidence and outcomes of ARDS. Pediatric ARDS (PARDS) occurs less frequently and carries lower mortality than adult ARDS [17]. The Pediatric Acute Respiratory Distress Syndrome Incidence and Epidemiology (PARDIE) study estimated that approximately 3% of patients in pediatric ICUs (PICUs) developed PARDS [18], while the incidence of neonatal ARDS in China was reported as 1.44% [19]. Consistent with this, a cohort study found a 90-day mortality rate of 19% in children, substantially lower than the 33% observed in adults [20], suggesting age contributes to outcome heterogeneity. Additionally, ARDS is more common in males than females [9, 21, 22]. Although Black patients may have a lower risk of developing ARDS [9], they appear to face significantly higher risks of adverse outcomes compared with White patients [23]. However, data from clinical trials present a more complex picture of racial disparities in ARDS outcomes [24, 25], which may be confounded by socioeconomic factors and biases in trial recruitment.

The recently proposed global definition of ARDS is reshaping its epidemiological landscape. The COVID-19 pandemic emerged as a major driver of increased ARDS incidence and mortality [2]. Notably, many patients with acute hypoxemic respiratory failure managed with high-flow nasal oxygen (HFNO) during the pandemic did not meet the Berlin definition criteria [26], profoundly affecting epidemiological estimates. The 2023 global definition enhances diagnostic inclusivity, particularly in resource-limited settings, and is likely to increase reported incidence. However, it does not address differences in long-term functional outcomes among ARDS survivors. Future prospective epidemiological studies using this new definition are needed to evaluate long-term prognosis. In conclusion, continuously refining our global understanding of ARDS epidemiology remains essential for accurately assessing morbidity, mortality, risk factors, and long-term outcomes.

## Etiology and host susceptibility

The evolving epidemiological landscape of ARDS highlights the need to better define not only its incidence and outcomes but also the factors that determine who develop the syndrome after an insult. The risk factors for the development of ARDS can be broadly classified into two categories: etiological insults (such as direct and indirect lung injury) and host susceptibility factors, including population-specific characteristics, environmental exposures, and comorbidities.

### Common precipitants and risk factors

**Fig. 1:**
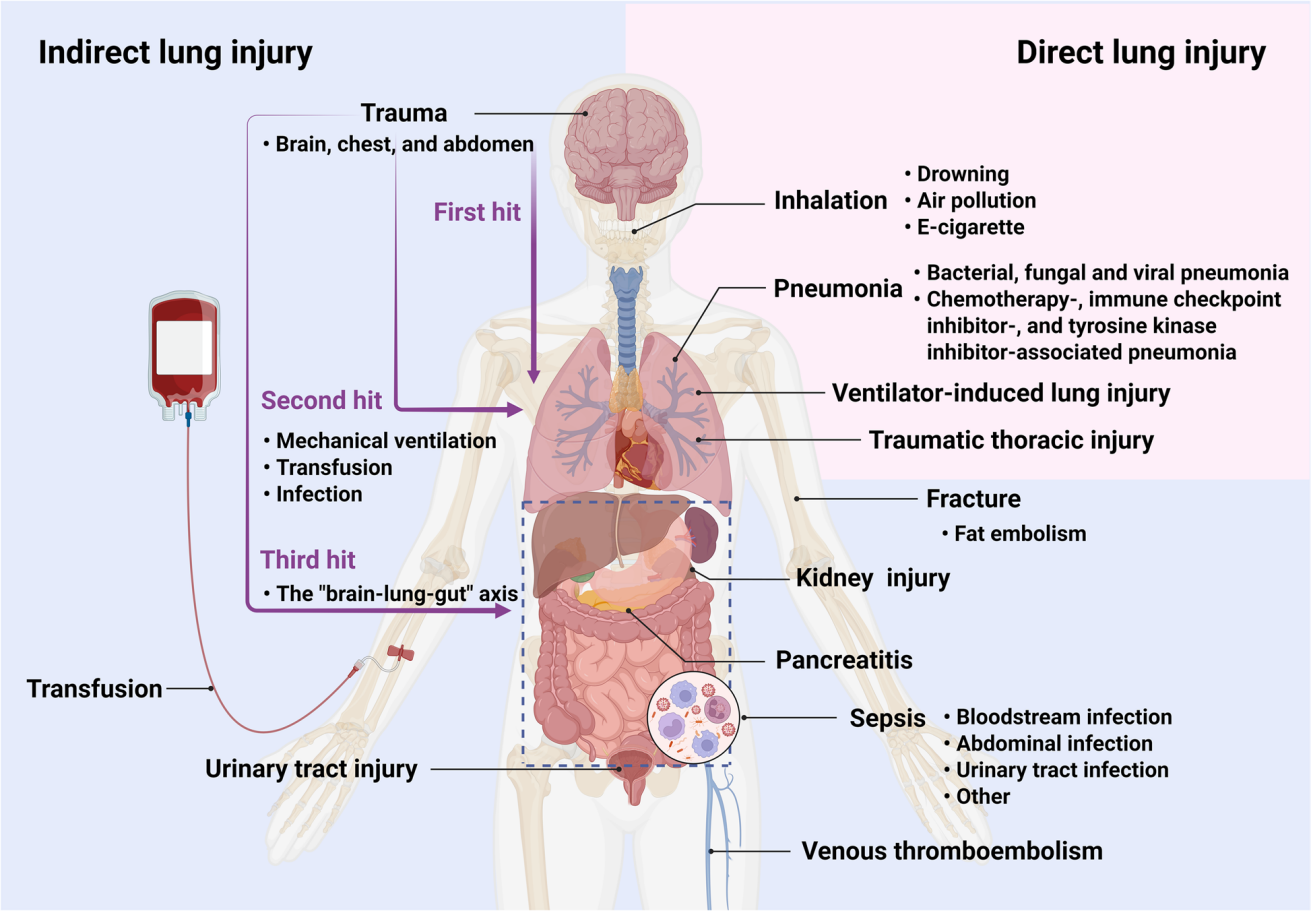
Etiology of acute respiratory distress syndrome (ARDS). Acute risk factors for ARDS are categorized into direct and indirect lung injury. Direct lung injury primarily includes pneumonia and inhalation injury, whereas indirect lung injury encompasses non-pulmonary infections (e.g., sepsis, pancreatitis), trauma, and transfusion. Among all contributing factors, infection is the most common cause. Using trauma as an example, the “triple hit” theory has been proposed

Acute risk factors for ARDS are broadly categorized as direct or indirect lung injury. Direct injury includes pneumonia [4, 5, 27–29], such as bacterial, fungal, and viral pneumonia [30], as well as drug-induced pneumonitis from chemotherapy, immune checkpoint inhibitors [31], or tyrosine kinase inhibitors [32], along with inhalation injury and ventilator-induced lung injury (VILI) [33]. Indirect injury encompasses non-pulmonary sources such as sepsis [14, 34] and pancreatitis [4], as well as trauma [35] and transfusion [36] (Fig. 1).

Among all etiologies, infection, both pulmonary and extrapulmonary, is the most common cause of ARDS. However, a causative pathogen remains unidentified in 30%–60% of cases [2], underscoring the need for rapid diagnostic approaches like metagenomic sequencing to guide targeted therapy and improve outcomes. Other less frequent causes include trauma (approximately 6% of ARDS cases) [35], pancreatitis (~3%), drowning (0.6%) [10], and transfusion-related acute lung injury (ALI), particularly associated with plasma from male donors, occurring in only 3–8 cases per million transfused units [36]. Immune checkpoint inhibitor pneumonitis (CIP) is another emerging etiology, with an incidence of 1.1%–6.4% in clinical trials [37]; real-world rates may be higher, and in nivolumab-induced CIP, ARDS accounts for about 10% of cases [31]. Trauma exemplifies the complex interplay of multiple insults in ARDS pathogenesis through the proposed “triple hit” theory [38]. First, trauma releases numerous inflammatory mediators into systemic circulation, establishing a pro-inflammatory state (“first hit”). Second, superimposed insults such as mechanical ventilation, infection, or blood transfusion deliver a “second hit”. Third, injuries to the brain, chest, or abdomen can disrupt gut microbiota via the “brain-lung-gut” axis, and this dysbiosis together with intestinal dysfunction constitutes a “third hit” [39, 40]. Consequently, effective trauma management requires a multidisciplinary approach that integrates gut microbiota modulation with comprehensive intensive care to develop individualized treatment strategies.

### Host susceptibility factors

Age, sex, race, ethnicity, and other population-specific characteristics are established risk factors for ARDS development. Environmental exposures, including air pollution [41, 42] and e-cigarette use [43, 44], further increase susceptibility. Comorbidities also influence ARDS risk and outcomes. For example, a multinational observational study showed that cancer patients with ARDS had higher 90-day mortality, irrespective of extracorporeal membrane oxygenation (ECMO) use [45]. The impact of obesity on ARDS prognosis remains complex: it may confer a survival advantage in sepsis-related ARDS [46, 47] but increases risk in SARS-CoV-2-associated ARDS [48].

Susceptibility to ARDS and its adverse outcomes arises from a highly complex interplay among etiology, host factors, and their interaction. This “etiology–host–mechanism” triad drives disease heterogeneity: the etiology determines the nature of injury, the host shapes the response pattern, and their interaction generates diverse trajectories of inflammation, immune activation, and repair, ultimately defining distinct phenotypes and prognoses. It is precisely this heterogeneity, spanning from etiological triggers to molecular mechanisms, that motivates deeper investigation into the core pathobiological pathways of ARDS, which we discuss next.

## Molecular mechanisms of ARDS

**Fig. 2:**
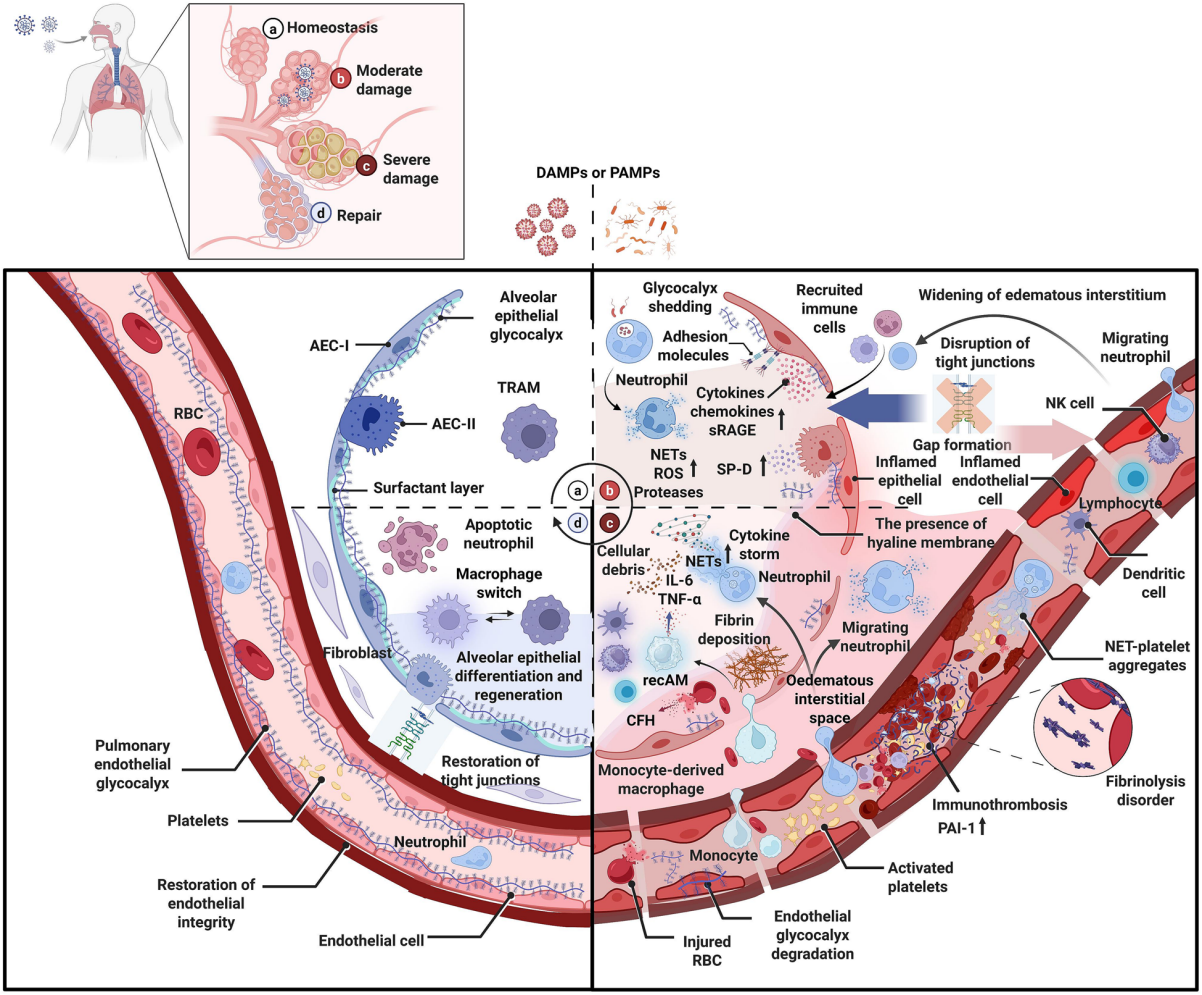
Model of pathogenesis of acute respiratory distress syndrome (ARDS). Homeostasis. The lung epithelium consists of type I (AEC I) and type II alveolar epithelial cell (AEC II), the latter producing surfactant. Both cell types actively transport fluid from the alveoli to maintain dry airspaces. The epithelial and endothelial glycocalyx layers and tight junctions remain intact, preserving barrier integrity. Moderate damage. Early bacterial or viral injury to the alveolar epithelium promotes protein-rich pulmonary edema, impairs surfactant production and alveolar fluid clearance, and initiates an inflammatory cascade. Damaged epithelial cells release adhesion molecules and chemokines, recruiting immune cells to the site of injury. Shedding of the epithelial and endothelial glycocalyx and disruption of tight junctions occur, with epithelial damage exacerbating endothelial injury. Severe damage. Severe disruption of the alveolar–capillary barrier leads to hyaline membrane formation and marked edema. Neutrophil extracellular traps (NETs) promote platelet aggregation, contributing to immunothrombosis. Coagulation abnormalities amplify the inflammatory response, and red blood cells (RBCs) crossing the barrier lyse, releasing cell-free hemoglobin (CFH) into the alveoli. Together with recruited neutrophils, macrophages, and lymphocytes, this induces oxidative stress and sustains persistent inflammation. Repair. This phase involves resolution of inflammation, clearance of alveolar edema, and restoration of the alveolar–capillary barrier. Neutrophils undergo apoptosis and are cleared; alveolar macrophages (AMs), along with chemokines and cytokines, shift from a pro-inflammatory to an anti-inflammatory phenotype. Alveolar epithelial cells regenerate and differentiate, the glycocalyx is restored, and reassembly of tight junctions supports alveolar fluid clearance. Fibroblasts contribute to normal tissue repair but may also drive pulmonary fibrosis. AEC I: type I alveolar epithelial cell; AEC II: type II alveolar epithelial cell; TRAM: tissue-resident alveolar macrophage; CFH: cell-free hemoglobin; NK cell: natural killer cell; AMs: alveolar macrophages; NETs: neutrophil extracellular traps; ROS: reactive oxygen species; sRAGE: soluble receptor for advanced glycation end products; SP-D: surfactant protein-D; PAI-1: plasminogen activator inhibitor-1; recAM: recruited alveolar macrophage; TNF-α: tumor necrosis factor-α; IL-6: interleukin-6; RBC: red blood cell

The pathogenesis of ARDS involves disruption of the alveolar–capillary barrier and a complex interplay of inflammatory, immune, and coagulation pathways that operate in both pulmonary and systemic compartments (Fig. 2). Core pathophysiological processes involve four interconnected components: (1) disruption of the alveolar–capillary barrier; (2) dysregulated inflammation, immunity, and coagulation; (3) impaired resolution, repair, and progression to fibrosis; and (4) an immune-mediated injurious microenvironment, further shaped by the spatiotemporal heterogeneity of the syndrome. This complexity highlights the urgent need for phenotype-based stratification to advance precision medicine in ARDS.

### Alveolar-capillary barrier disruption

The alveolar–capillary barrier, composed of alveolar epithelial and capillary endothelial cells, is central to the characteristic physiological and clinical features of ARDS. Circulating pathogens and other predisposing factors induce injury to the alveolar epithelium, particularly type II alveolar epithelial cell (AEC II), reducing surfactant production [49]. This impairs alveolar fluid clearance and promotes pulmonary edema [50], thereby initiating pro-inflammatory and pro-coagulant cascades.

Alveolar epithelial cell necrosis and leakage of intracellular contents release damage-associated molecular patterns that amplify inflammatory signaling, driving cytokine and chemokine expression to recruit immune cells to the site of injury. Concurrently, degradation of the alveolar epithelial glycocalyx contributes to surfactant dysfunction and ARDS pathogenesis [51]. In addition, injured alveolar epithelial cells release tissue factor (TF) and shed anticoagulant molecules, facilitating fibrin deposition [52] and hyaline membrane formation within the alveoli, a hallmark observed in the original 1967 autopsy report of ARDS patients [53]. The alveolar epithelium also functions as a critical biological barrier against infection [54]; its disruption permits pathogen invasion.

Epithelial injury simultaneously exacerbates capillary endothelial damage. Under stimulation by endogenous signals and pro-inflammatory cytokines, endothelial cells become activated and exhibit multiple functional abnormalities [55, 56], including glycocalyx degradation [57], enhanced leukocyte adhesion and recruitment [58], coagulation dysregulation [59], increased vascular permeability [60], impaired vasodilation and angiogenesis, and oxidative stress imbalance [61]. Glycocalyx loss exposes [57] endothelial adhesion molecules such as P- and E-selectins, promoting platelet and leukocyte adhesion to the endothelial surface. This facilitates transendothelial migration of immune cells into the alveolar space [62], further amplifying inflammation [63] and oxidative stress. Excessive production of reactive oxygen species (ROS) can induce endothelial cell death and disrupt intercellular tight junctions [61]. The resulting increase in vascular permeability [60] allows protein-rich fluid to leak into the interstitial and alveolar compartments, causing edema and perpetuating procoagulant and inflammatory cascades.

In summary, epithelial and endothelial injuries reciprocally reinforce one another, establishing a vicious cycle in which barrier disruption serves both as an initial trigger and a key driver of ARDS progression. Consequently, strategies to mitigate injury and restore an intact alveolar–capillary barrier represent a major focus of current ARDS research.

### Dysregulated inflammation, immune, and coagulopathy

#### Inflammation and immune dysregulation

The inflammatory cascade and immune dysregulation initiated by alveolar–capillary barrier disruption lie at the core of ARDS pathogenesis. Immune responses in ARDS involve not only innate and adaptive immunity but also non-immune circulating cells, particularly red blood cells and cell-free hemoglobin (CFH), which actively contribute to inflammation [64, 65].

Following barrier breakdown, protein-rich fluid and inflammatory cells flood the interstitium and alveoli, promoting recruitment of immune effectors such as neutrophils to sites of tissue injury [61]. Activated neutrophils adopt a pro-inflammatory and procoagulant phenotype, releasing neutrophil extracellular traps (NETs) [61, 66, 67]. NETs induce necroptosis in alveolar epithelial cells [68], enhance platelet aggregation and activation [60], and release proteases, ROS, and pro-inflammatory lipid-derived mediators, including leukotrienes (LTs) and prostaglandins (PGs) [2], thereby exacerbating lung tissue damage.

Macrophages detect damage-associated and pathogen-associated molecular patterns (DAMPs and PAMPs) through pattern recognition receptors (PRRs), driving pro-inflammatory polarization and the secretion of cytokines and chemokines [69, 70]. Notably, our group demonstrated that macrophage-specific deletion of phosphatase and tensin homolog deleted on chromosome 10 (PTEN) upregulates nuclear factor E2-related factor 2 (NRF2) expression and alleviates lipopolysaccharide-induced ALI by inhibiting the Hippo–YAP signaling pathway [71]. More recently, we found that Jagged1-mediated crosstalk between the macrophage Notch1/Foxo1 signaling axis regulates the thioredoxin-interacting protein (TXNIP)/NOD-, LRR- and pyrin domain-containing protein 3 (NLRP3) inflammasome, thereby mitigating pulmonary inflammation [72].

Beyond neutrophils and macrophages, other immune cells, including lymphocytes, dendritic cells, and natural killer (NK) cells, also contribute to the inflammatory milieu in ARDS [73]. Additionally, red blood cells that cross the disrupted barrier undergo lysis, releasing CFH into the alveolar space. CFH promotes oxidative stress and amplifies inflammatory responses. Clinical studies have identified CFH as a significant predictor of mortality in patients with severe sepsis [74] and as an independent risk factor for ARDS development [75].

Collectively, the immune landscape in ARDS is characterized by intricate crosstalk among professional immune cells, injured structural cells, and systemic mediators such as CFH, which together establish a self-amplifying pro-inflammatory loop. This complexity underscores the therapeutic potential of targeting specific molecular pathways, including the PTEN/YAP and Notch1/Foxo1 axes identified in our work, as well as modulating systemic redox balance, to develop novel immunomodulatory strategies for ARDS.

#### Immunothrombosis and coagulopathy

Immune dysregulation in ARDS drives excessive immunothrombosis, leading to a profound imbalance between coagulation and fibrinolysis. Immunothrombosis is primarily initiated by monocytes and neutrophils, with additional contributions from complement activation and endothelial cell activation [76].

Complement activation amplifies the platelet–neutrophil extracellular trap (NET)–TF–thrombin axis, promoting immunothrombus formation. Cleavage of complement components generates C3a and C5a: C3a can activate platelets, whereas C5a, along with platelet-derived thrombin, induces neutrophils to express TF and release NETs decorated with active TF, thereby triggering the extrinsic coagulation pathway. These pro-thrombotic NETs further stimulate endothelial cells to upregulate TF expression, enhancing their pro-coagulant activity [77]. Consequently, pharmacologic inhibition of C5aR1, thrombin, or NET formation attenuates platelet-mediated, NET-driven thrombosis [22, 77], highlighting complement, NETs, and thrombin as promising therapeutic targets in ARDS.

NETs also promote coagulopathy through multiple mechanisms. They directly activate factor XII, initiating the intrinsic coagulation cascade [76]. Moreover, NETs contain DNA complexed with histones and antimicrobial proteins such as neutrophil elastase (NE) and myeloperoxidase (MPO) [78]. NE and MPO cleave and inactivate key natural anticoagulants, including thrombomodulin (TM) and tissue factor pathway inhibitor (TFPI) [76], while histones enhance thrombin generation by suppressing thrombomodulin-dependent protein C activation [79] and by activating platelets [60, 80]. Additionally, von Willebrand factor (VWF), which participates in NET formation [81], binds directly to histones. VWF not only serves as a major adhesive protein mediating platelet–vessel wall interactions but also facilitates platelet recruitment [61].

Activated endothelial cells further propagate the coagulation cascade. Shedding of surface anticoagulant molecules coupled with upregulation of procoagulant factors promotes microvascular thrombus formation in injured regions, obstructing the pulmonary vascular bed. This increases dead space ventilation and contributes to pulmonary arterial hypertension [4]. Concurrently, impaired fibrinolysis exacerbates coagulopathy in ARDS. Patients exhibit markedly elevated levels of fibrinolytic inhibitors, particularly plasminogen activator inhibitor-1 (PAI-1) [82], which correlate with disease severity and further suppress fibrinolysis, potentially accelerating coagulation dysfunction [83].

Critically, thrombin generation and platelet activation not only sequester immune cells but also amplify inflammation by stimulating additional cytokine release [63], establishing a self-reinforcing inflammation–coagulation feedback loop.

In summary, ARDS pathogenesis involves excessive immunothrombosis in pulmonary microvessels, driven by dynamic crosstalk between the immune and coagulation systems. This interdependence suggests that dual-targeted interventions simultaneously modulating inflammation and coagulation may offer novel therapeutic avenues for ARDS.

### Impaired resolution, repair, and fibrosis

Resolution of ARDS entails the attenuation of inflammation, clearance of alveolar edema, and restoration of the alveolar–capillary barrier. This phase involves coordinated inflammatory resolution and structural repair, with fibroblasts playing dual roles in both tissue regeneration and pathological fibrosis [55, 84]. The transition from acute injury to resolution represents a tightly regulated balance between pro-inflammatory and pro-resolving signals. Failure to effectively resolve inflammation and reestablish barrier integrity predisposes the lung to fibroproliferative remodeling and adverse clinical outcomes.

### Immune-mediated damage microenvironment and spatiotemporal heterogeneity of ARDS

#### Immune-mediated damage microenvironment in ARDS

The interplay among the host microecology, immunity (including therapeutic interventions), and tissue injury shapes an immune-mediated damage microenvironment that critically influences ARDS onset and progression. Pathogen-induced infections can trigger ARDS and ALI, with subsequent immune dysregulation manifesting either as excessive inflammation causing collateral organ damage or as immunosuppression [85] that heightens susceptibility to secondary infections. Latent viruses, such as cytomegalovirus (CMV), Epstein–Barr virus (EBV) [86], or herpes simplex virus (HSV) [87], as well as colonizing bacteria [88] and fungi [89] within the host microecology may reactivate under these conditions, serving as persistent drivers of tissue injury and inflammation in ARDS. The highly inflammatory milieu of ARDS not only fosters the proliferation of fungi like Aspergillus [90] but also renders patients more vulnerable to invasive fungal infection, particularly when corticosteroid therapy further compromises antifungal immunity. In this context, neutrophils utilize NETs to capture and clear Aspergillus fumigatus [91]; however, these same NETs exacerbate ARDS and multiorgan dysfunction by promoting dysregulated immunothrombosis [76].

Managing such immune dysregulation remains challenging. No gold standard exists for diagnosing viral reactivation, and viral loads vary significantly across sampling sites [87], with no clinically validated threshold defining pathogenic significance. Moreover, current diagnostic methods cannot reliably distinguish active bacterial or fungal infection from mere colonization. Consequently, the benefit of antimicrobial prophylaxis in high-risk patients with unresolved ARDS remains uncertain. Critically, substantial interpatient heterogeneity in both inflammatory intensity [92] and the degree of immunosuppression results in highly divergent host response trajectories. This continuum spans from an initial phase of immune resistance, aimed at reducing pathogen burden, to disease tolerance [93], a state in which the host actively limits ARDS severity by preserving tissue and barrier integrity, and ultimately to immune resilience [63], defined as the capacity of the immune system to return to its pre-ARDS homeostatic state, ultimately in inflammation resolution. A deeper understanding of the dynamic interactions among microecology, host immunity, and tissue damage within this immune-mediated damage microenvironment, and moving beyond the oversimplified dichotomy of hyperinflammation versus immunosuppression, is therefore essential for improving ARDS management and patient outcomes.

#### Temporal heterogeneity

Immune dysregulation in ARDS exhibits marked temporal heterogeneity. The inflammatory response no longer conforms to the traditional two-stage model, initial hyperinflammation followed by sustained immunosuppression, but instead reflects a multidimensional state in which pro- and anti-inflammatory pathways are simultaneously activated and intricately intertwined [63]. Consequently, the optimal timing for interventions targeting inflammation or immunosuppression in ARDS remains controversial.

#### Immune compartmentalization

Previous analyses of host responses in ARDS have largely relied on blood-derived biomarkers, yet systemic immune activity may differ substantially from local responses in the lungs. Current evidence indicates that systemic and alveolar inflammatory responses in ARDS follow distinct developmental trajectories [94, 95], reflecting compartmentalized inflammation [63, 95–97]. Alveolar inflammation may spread from the lung compartment to systemic circulation, originate from systemic inflammation seeding the alveoli, or arise concurrently in both compartments, resulting in distinct inflammatory endotypes [4]. For instance, ARDS associated with COVID-19 exhibits an immunophenotype characterized by peripheral neutrophilia and lymphopenia, but often with a relatively milder systemic inflammatory response compared to classic ARDS [2]. This compartmentalization complicates immune-targeted therapies guided solely by circulating biomarkers, as plasma inflammatory levels may not accurately reflect the burden within the airways, and the association between individual biomarkers and patient prognosis can vary depending on the sampling compartment, likely explaining the heterogeneous treatment responses observed in trials of immunomodulation based exclusively on blood measurements.

In summary, immune dysregulation and spatiotemporal heterogeneity drive the phenotypic diversity of ARDS. Advancing precision medicine will require a deeper understanding of host response mechanisms and theragnostic biomarkers to enable robust phenotypic stratification [98], thereby differentiating patients not only by treatability but also by treatment response and prognosis [99]. Achieving such phenotype-based interventions, however, first demands a unified clinical standard for accurately identifying and defining ARDS subtypes, a need that remains the cornerstone driving the continuous evolution of ARDS diagnostic criteria.

## Clinical diagnosis and assessment

Accurate diagnosis and structured clinical assessment are essential for the management of ARDS, as the syndrome is defined by clinical, pathophysiological, and imaging criteria rather than by a single specific diagnostic test. This section will first review the historical evolution of ARDS diagnostic standards (including the Berlin Definition and 2023 global consensus) before dissecting the diagnostic performance of conventional radiology, lung ultrasound and artificial intelligence (AI)-assisted imaging. We also highlight key trade-offs between diagnostic accessibility for under-resourced medical centers and the preservation of robust specificity, reproducibility and clinical practicability.

### Evolution of diagnostic criteria

**Fig. 3:**
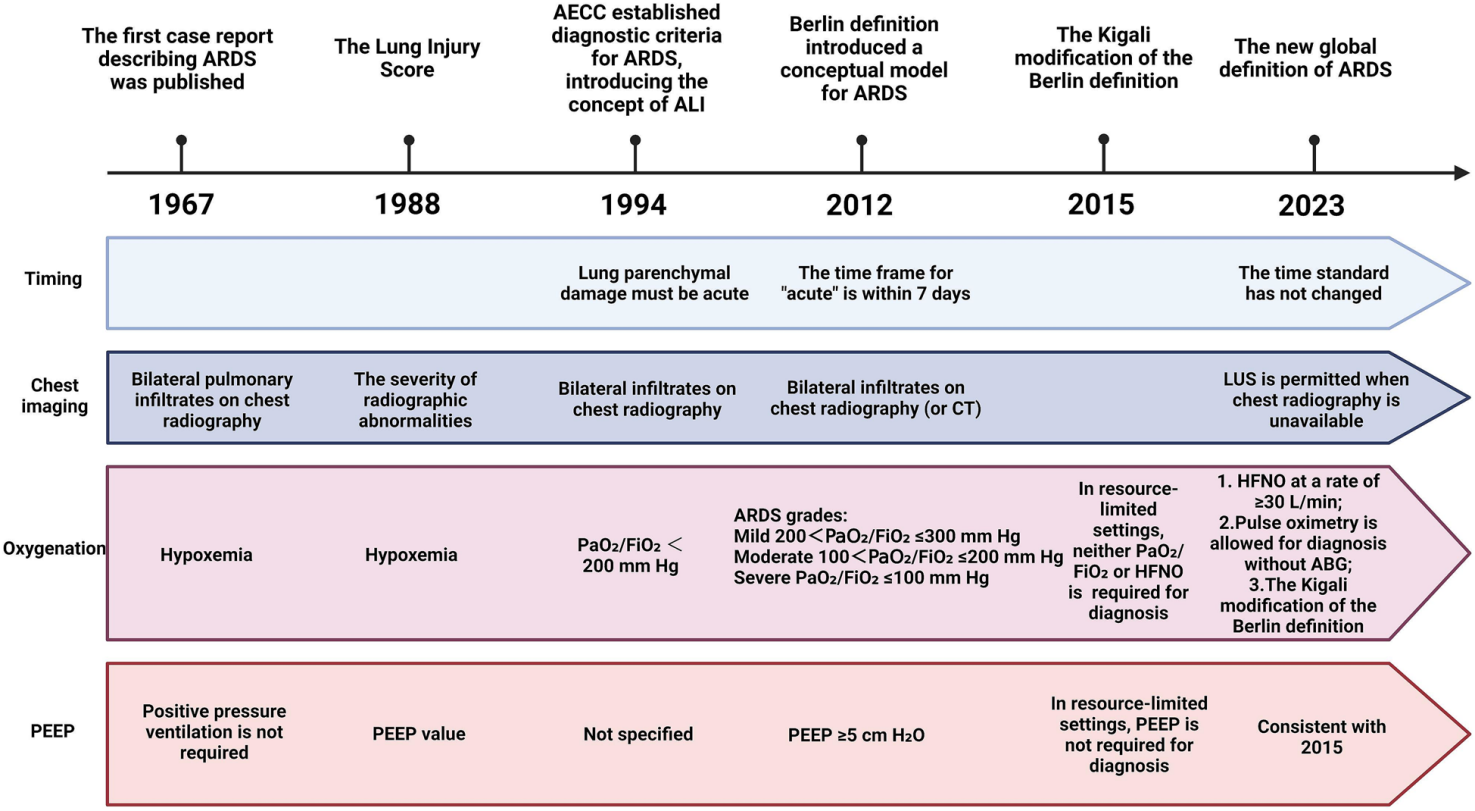
Evolution of the diagnostic criteria for acute respiratory distress syndrome (ARDS). This figure outlines key milestones in the evolution of ARDS diagnostic criteria across four domains: time, chest imaging, oxygenation, and positive end-expiratory pressure (PEEP). AECC: American-European Consensus Conference; ALI: Acute lung injury; CT: Computed tomography; LUS: Lung ultrasound; PaO₂: Partial pressure of arterial oxygen; FiO₂: Fraction of inspired oxygen; HFNO: High-flow nasal oxygen; ABG: Arterial blood gas; PEEP: Positive end-expiratory pressure

Research into ARDS has driven a progressive refinement of its definition (Fig. 3). The first case report describing ARDS appeared in 1967 [53], noting common features such as tachypnea, hypoxemia, and reduced lung compliance. Bilateral pulmonary infiltrates were evident on chest radiography, closely resembling hydrostatic pulmonary edema, though positive pressure ventilation was not included as a defining criterion.

The Lung Injury Score, proposed in 1988 [100], incorporated radiographic severity, hypoxemia, positive end-expiratory pressure (PEEP) in mechanically ventilated patients, and lung compliance (if measured). A score exceeding 2.5 defined ARDS, even without measurements of positive pressure ventilation or compliance. In 1994, the American-European Consensus Conference (AECC) established formal diagnostic criteria for ARDS, introducing the term “ALI” and requiring acute onset of parenchymal lung damage [101]. The 2012 Berlin definition introduced a conceptual framework that specified an “acute” timeframe of within 7 days, eliminated ALI, and classified ARDS into mild, moderate, and severe categories, mandating mechanical ventilation, either invasive or non-invasive, with PEEP ≥ 5 cm H₂O [15].

Accurate and timely ARDS diagnosis remains challenging, particularly in resource-limited settings. The 2023 global definition [1] aims to facilitate earlier identification, diagnosis, and intervention while ensuring inclusion of patients in such settings. Key updates include: (1) permitting lung ultrasound when chest radiography is unavailable; (2) including patients receiving HFNO at ≥ 30 L/min; (3) allowing pulse oximetry for diagnosis without arterial blood gas (ABG), using SpO₂/FiO₂ for severity assessment when SpO₂ ≤ 97%; (4) establishing a new non-intubated ARDS category; and (5) incorporating the Kigali modification of the Berlin definition, which enables diagnosis in resource-limited contexts without PaO₂/FiO₂, PEEP, or HFNO requirements.

Despite improving diagnostic inclusivity, the new definition has limitations [102–104]. First, it may reduce specificity: pulse oximetry can be affected by skin pigmentation, shock, or poor perfusion [105], warranting ABG confirmation when feasible. Second, it risks increasing diagnostic inconsistency, as the validity of thresholds for HFNO flow rate and SpO₂/FiO₂, along with operator competency and ultrasound availability in resource-limited settings, remains debated, potentially elevating false-positive rates. Finally, current ARDS subphenotypes, particularly hyperinflammatory and hypoinflammatory types identified through plasma biomarker and clinical data clustering, are not yet integrated into the definition. These subgroups are expected to be incorporated once they demonstrate differential treatment responses [106]. Consequently, this global definition may not be final and requires validation in large patient cohorts to assess its effectiveness, validity, and reliability.

### Imaging in ARDS diagnosis

#### Conventional imaging in ARDS diagnosis

Chest imaging is a defining component of ARDS diagnosis. Bilateral pulmonary infiltrates on chest radiography represent a key radiographic criterion. However, analysis of the LUNG-SAFE study showed that patients with unilateral infiltrates had mortality rates similar to those with bilateral infiltrates [107], suggesting that early interventions, such as lung-protective ventilation, may be warranted [3]. The prognostic implications and clinical relevance of unilateral versus bilateral opacities on chest radiography require further evaluation.

Conventional imaging modalities, while indispensable in ARDS assessment, have notable limitations. Chest radiography is widely accessible but lacks sensitivity for early disease detection, whereas chest computed tomography (CT) provides detailed visualization of lung lesions at the cost of radiation exposure and risks associated with patient transport [108, 109]. Recognition of these shortcomings has spurred interest in more objective, quantitative approaches, such as lung ultrasound and artificial intelligence (AI)-assisted image analysis, which may enhance the accuracy and timeliness of ARDS diagnosis in the future.

#### Lung Ultrasound (LUS)

The new global definition of ARDS includes lung ultrasound (LUS) as an alternative imaging modality [1]. LUS enables bedside, real-time, and repeatable assessment of lung morphology [110]. A multicenter prospective observational study, the Application of Lung Ultrasound in ARDS and its Validation against Chest Radiography, demonstrated that a data-driven LUS-ARDS score can accurately diagnose ARDS, even after external validation [111]. This score relies on identifying B-lines (indicative of alveolar-interstitial syndrome) and consolidations, both hallmark features of ARDS.

Despite these promising findings, several limitations affect the generalizability of current LUS diagnostic models. First, most patients in the study had ARDS secondary to pulmonary causes; it remains unclear whether the model applies to extrapulmonary ARDS (e.g., from sepsis or pancreatitis), which often presents with distinct radiographic patterns. Second, the ultrasound operators in the study were highly trained, a level of expertise not always available in routine practice, highlighting the need for adequate training when using LUS for ARDS diagnosis. Additionally, technical challenges such as subcutaneous emphysema, pleural effusions, and obesity can impair acoustic windows and reduce diagnostic accuracy.

Nevertheless, the portability and non-invasiveness of LUS make it invaluable for initial assessment and serial monitoring of ARDS, particularly in resource-limited settings or for unstable patients who cannot undergo CT. Future efforts should focus on developing simplified, universally applicable scoring systems and integrating AI-assisted image analysis to minimize operator dependency and further improve diagnostic precision.

#### Artificial intelligence (AI)—assisted imaging in ARDS diagnosis

Artificial intelligence (AI) offers a promising approach to addressing the subjectivity and variability inherent in conventional imaging. Deep learning algorithms can automatically analyze medical images, enabling rapid, standardized, and consistent quantification of lung abnormalities. A multicenter cohort study [112] demonstrated that a deep learning framework based on the UNet Transformer (UNETR) model achieved high accuracy and robustness in segmenting lung lesions and predicting early ARDS, with strong generalizability and clinical applicability. In addition, some studies [113] have developed open-source tools that apply machine learning models, such as the eXtreme Gradient Boosting (XGBoost), to automatically detect bilateral pulmonary infiltrates from radiology reports, integrating physician notes to identify ARDS cases. As AI and machine learning continue to advance, the efficiency of chest imaging–based ARDS diagnosis is expected to improve substantially [114]. Beyond diagnostic support, AI may also generate novel hypotheses to guide future data collection and experimental design, thereby deepening our understanding of ARDS pathophysiology.

Nevertheless, AI-assisted imaging faces significant challenges related to data heterogeneity and clinical interpretability. Key limitations include small sample sizes, insufficient external validation, and dependence on image quality. Moreover, model performance often varies considerably across different populations and clinical settings. Future research should prioritize multi-center data integration for collaborative model training, leveraging diverse cohorts to enhance robustness and real-world applicability. Equally important is the incorporation of expert clinical knowledge into model development to ensure clinical relevance and foster effective human–AI collaboration, ultimately improving predictive accuracy and generalization.

Taken together, current approaches to the clinical diagnosis and assessment of ARDS still require validation across diverse healthcare settings. This challenge highlights the importance of developing more precise and clinically applicable diagnostic and therapeutic frameworks. Biomarker-based phenotypic stratification may therefore provide a promising pathway toward precision medicine in ARDS and serves as a foundation for the following discussion.

## From biomarkers to phenotypic stratification of ARDS

Phenotypic stratification guided by biomarkers represents a key strategy for overcoming the current challenges in ARDS management. Given the substantial epidemiological differences between PARDS and adult ARDS, as well as the biological variations in lung development and maturation from infancy through adulthood [17], precise characterization of ARDS across diverse populations is critically important. To address this heterogeneity, computational algorithms are used to cluster physiological, clinical, or molecular data and identify more homogeneous subgroups, termed subphenotypes. When these subphenotypes reflect distinct pathophysiological mechanisms that lead to differential responses to targeted therapies, they are further classified as endotypes [6, 63, 70, 115].

Within this precision medicine framework, two enrichment strategies are central: prognostic enrichment, which selects patients at higher risk for clinically meaningful outcomes, and predictive enrichment, which identifies those more likely to respond to a specific therapy based on its mechanism of action [116]. The ultimate goal is to deliver the right treatment, tailored to individual patients (e.g., those with PARDS or adult ARDS), at the optimal dose and timing. However, a core challenge remains the development of clinically relevant biomarkers and their effective combinations.

### Biomarkers

Individual biomarkers encompass blood cell markers, soluble receptors, acute-phase proteins, gene transcripts, cytokines, and metabolites [63]. Inflammatory mediators associated with ARDS include tumor necrosis factor (TNF), interleukin (IL)-1, IL-6, IL-8 [117], and ferritin. Additional categories include markers of dysregulated coagulation (e.g., PAI-1 and protein C), endothelial injury (e.g., angiopoietin-2 [Ang-2] [118] and intercellular adhesion molecule-1), and epithelial injury (e.g., soluble receptor for advanced glycation end products [sRAGE] [119] and surfactant protein-D [SP-D] [120]). These biomarkers have been linked to ARDS pathogenesis and prognosis and may represent potential therapeutic targets [121–123], though large-scale clinical validation is still needed.

### Using biomarkers and their combinations for subgroup identification in ARDS

**Fig. 4:**
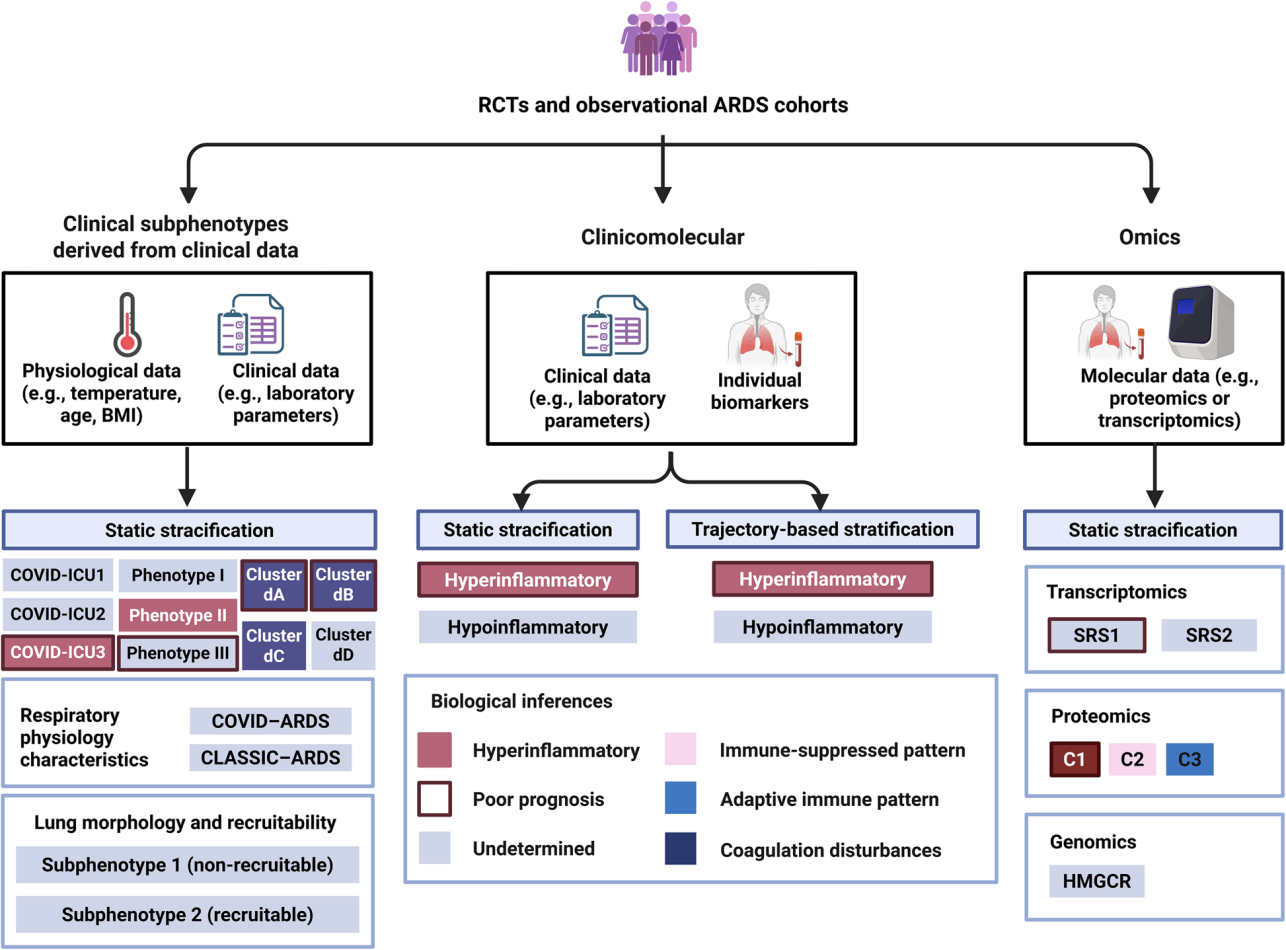
Precision phenotyping in acute respiratory distress syndrome (ARDS). Given the marked heterogeneity of ARDS, research has focused on identifying subgroups through integration of physiological (e.g., temperature, age, BMI), clinical (e.g., laboratory parameters), and molecular data (e.g., proteomics, transcriptomics). Clustering techniques have been applied to both single-timepoint data (static stratification) and longitudinal profiles (trajectory-based stratification). The resulting subgroups are associated with distinct biological signatures, including inflammation, immune dysregulation, and coagulopathy, as well as differential outcomes such as mortality. A phenotype-oriented diagnostic and stratification framework can capture the clinical and biological complexity of ARDS and support personalized treatment strategies to improve patient outcomes. RCTs: Randomized controlled trials; BMI: Body mass index; SRS: Sepsis response state

Owing to the marked heterogeneity among ARDS patients, research has focused on integrating physiological data (e.g., temperature, age, body mass index [BMI]), clinical variables (e.g., laboratory parameters), and molecular profiles (e.g., proteomics, transcriptomics) to define subgroups (Fig. 4). These subgroups are associated with distinct biological signatures, including inflammation, immune dysregulation, and coagulopathy, as well as differential outcomes such as mortality [63, 124, 125]. By establishing a phenotype-oriented diagnostic and stratification framework, this approach captures the clinical and biological complexity of ARDS and enables personalized interventions to improve patient outcomes.

#### Clinical subphenotypes derived from clinical data

Clinically relevant subphenotypes can be identified using conventional clinical data without requiring novel biomarker measurements. In one study, machine learning applied to routine clinical parameters from critically ill patients with COVID-19 revealed three distinct clinical phenotypes with differing immunological profiles. Among these, the hyperinflammatory phenotype, designated COVID-ICU3 and characterized by advanced age, a high proportion of males, multiple comorbidities, acute kidney injury, and metabolic disturbances, was associated with the highest mortality rate; notably, corticosteroid treatment was not linked to worse survival in this group [126].

Another study [127] leveraged data from the ALVEOLI, FACTT, and SAILS randomized controlled trials (RCTs) to derive three clinical ARDS phenotypes based on baseline characteristics and prognosis, aiming to uncover differential treatment responses. Additional subphenotypes have been proposed based on trajectories of respiratory physiology [128] or on lung morphology and recruitability [3, 129, 130]. Moreover, clustering analyses incorporating coagulation markers, platelet counts, prothrombin time, and international normalized ratio have identified pronounced coagulopathic subgroups that may inform targeted anticoagulant strategies [131].

Collectively, clinical subtyping using routinely collected parameters offers a practical and cost-effective means to address ARDS heterogeneity, complementing biomarker-driven approaches in the pursuit of precision medicine.

#### Clinicomolecular

Integrating clinical data with plasma protein biomarkers provides another effective strategy for patient stratification. This clinicomolecular approach has consistently identified hyperinflammatory and hypoinflammatory subphenotypes in ARDS [92], which are now recognized as critical for both prognostic and predictive enrichment [70, 132–134]. Patients with the hyperinflammatory subphenotype appear to benefit from simvastatin [135], higher PEEP [136], and conservative fluid management [137]. In contrast, corticosteroids may exert detrimental effects in those with the hypoinflammatory subphenotype [138]. This divergent response to therapy underscores the necessity of clinicomolecular stratification to avoid “one-size-fits-all” interventions that could prove ineffective or harmful in specific subgroups.

#### Omics

Omics technologies, including genomics, proteomics, and metabolomics, hold promise for advancing ARDS understanding by uncovering disease-specific biomarkers and enabling personalized interventions [139–141]. Metagenomic analysis revealed a strong, albeit incomplete, overlap between the hyperinflammatory and hypoinflammatory ARDS phenotypes and the sepsis response signature 1 and SRS 2 endotypes [138]. Similarly, a secondary analysis of the ROSE (Reevaluation of Systemic Early Neuromuscular Blockade) trial demonstrated discordance between plasma protein concentrations and corresponding gene transcript levels, suggesting that transcriptomics- and proteomics-based approaches may define biologically distinct phenotypes with only moderate concordance [142]. This partial overlap highlights the need to integrate diverse phenotypic classification systems and further elucidate the underlying biological mechanisms of ARDS subphenotypes.

In a large-scale proteomic study, three reproducible ARDS subphenotypes, C1, C2, and C3, were identified and validated, each exhibiting distinct clinical, imaging, and molecular features [143]. C1 displayed robust innate immune activation and metabolic reprogramming, whereas C2 showed features of immunosuppression and restoration of anti-inflammatory metabolic pathways. C3 represented an intermediate state, with moderate immune activation and partial resolution of inflammation.

Additionally, a genome-wide association study [141] comparing ARDS patients with high-risk controls identified a common genetic variant near HMGCR associated with increased ARDS susceptibility, implicating cholesterol metabolism in disease pathogenesis. Together, these findings indicate that omics approaches can reveal novel biomarkers with therapeutic relevance. However, accurately defining ARDS subgroups will likely require integration of multiple analytical platforms, combining different omics modalities with emerging biomarkers, to capture the full biological complexity of the syndrome.

#### Dynamic phenotyping

Clustering techniques have been applied not only to single-timepoint data (static stratification) but also to longitudinal data (trajectory-based stratification), opening new avenues for precision medicine in ARDS. One study developed an open-source AI clinical classifier to evaluate the temporal stability of ARDS inflammatory phenotypes over 30 days and to assess how their evolution influences responses to corticosteroid therapy [144]. The hyperinflammatory phenotype demonstrated temporal instability, whereas the hypoinflammatory phenotype remained relatively stable. Glucocorticoid treatment was effective in the hyperinflammatory group but showed no benefit in the hypoinflammatory group [144], highlighting the therapeutic importance of tracking phenotypic evolution.

These findings underscore two key principles for precision intervention: (1) identifying phenotypes that respond to specific therapies, and (2) delivering the optimal treatment at the right time and dose. Similar patterns of phenotypic dynamics and their impact on treatment response and clinical outcomes have been confirmed in other ARDS studies, including those focused on PARDS [120, 142, 145]. Notably, PARDS research may also consider the influence of developmental age on phenotypic expression [17], as adult phenotyping paradigms do not fully translate to pediatric populations. This reinforces the need to define the distinct clinical and biological features of PARDS.

Collectively, these studies provide a strong theoretical foundation for using trajectory-based stratification in ARDS phenotyping, the approach with the greatest potential to date for evaluating candidate therapies in precision medicine.

Phenotypic stratification based on biomarkers has not only deepened our understanding of ARDS heterogeneity but also driven innovations in treatment strategies that are dynamic and individualized, offering a critical decision-making framework for the precise application of evolving ARDS therapies.

## Evolving therapeutic strategies

The increasing recognition of clinical and biological heterogeneity in ARDS has reshaped the therapeutic framework of the syndrome. Beyond foundational supportive care, corticosteroid therapy, and management of the underlying cause, growing attention has been directed toward cell-targeted and phenotype-guided strategies, including approaches aimed at inflammopathic and coagulopathic phenotypes. This section outlines current and emerging therapeutic strategies for ARDS and highlights how mechanistic insights and biological stratification may support the development of more individualized treatment approaches.

### Foundational supportive care

In the comprehensive management of ARDS, evidence-based supportive care is the cornerstone of improving patient outcomes and must be individualized. Interventions proven to improve survival include lung-protective ventilation with low tidal volumes (4–8 mL/kg predicted body weight) and plateau pressure limited to < 30 cm H₂O [7, 146], prone positioning for moderate-to-severe ARDS [147, 148], and conservative fluid management [149]. Table 1 summarizes areas of consensus and discordance between the American Thoracic Society (ATS) and European Society of Intensive Care Medicine (ESICM) clinical practice guidelines for ARDS.

**Table 1:** Consensus and discordance between the ATS and ESICM ARDS guidelines

	Intervention		Discordance	
			ATS	ESICM
Consensus	Low tidal volume ventilation	Strong for 4–8 mL/kg predicted body weight	Strong for inspiratory pressures (plateau pressure < 30 cm H_2_O)	-
	Recruitment maneuvers	Strong recommendation against prolonged recruitment maneuvers	-	Against routine use of brief high-pressure recruitment maneuvers (Weak recommendation)
	Prone positioning	-	Strong for severe ARDS	1. Strong for moderate-to-severe ARDS 2. Awake prone positioning for non-intubated patients with COVID-19 AHRF (Weak recommendation)
	VV-ECMO	The use of VV-ECMO for severe ARDS	Conditional for selected patients with severe ARDS	Strong for severe ARDS patients who meet the EOLIA trial inclusion criteria
Discordance	The ventilatory management of non-intubated patients with ARDS/AHRF	-	Not addressed	Strong for the use of HFNO over COT to reduce the risk of intubation in this population
	Corticosteroids	-	Conditional recommendations in favor of corticosteroids in ARDS	Not addressed
	Higher PEEP	-	Conditional for moderate-to-severe ARDS	No recommendation for or against use of high PEEP/FiO_2_ strategy in ARDS
	Neuromuscular blockade	-	Conditional for early (< 48 h since ARDS onset), severe (PaO_2_/FiO_2_ ratio ≤ 100 mmHg) ARDS	Strong against for routine use in moderate-to-severe ARDS
	Oscillatory ventilation	-	Strong recommendation against the routine use of high-frequency oscillatory ventilation in patients with moderate or severe ARDS	Not addressed
	Extracorporeal CO_2_ removal	-	Not addressed	Strong recommendation against extracorporeal CO_2_ removal

Based on the summary of guidelines on ARDS from the American Thoracic Society (ATS) [7] and the European Society of Intensive Care Medicine (ESICM) [6]

*ATS* American Thoracic Society, *ESICM* European Society of Intensive Care Medicine, *ARDS* acute respiratory distress syndrome, *AHRF* acute hypoxemic respiratory failure, *VV-ECMO* veno-venous extracorporeal membrane oxygenation, *HFNO* high-flow nasal oxygen, *COT* conventional oxygen therapy, *PEEP* positive end-expiratory pressure, *PaO₂* partial pressure of arterial oxygen, *FiO₂* fraction of inspired oxygen

#### Low tidal volume ventilation

Lung-protective ventilation is a key component in ARDS management, but simply reducing tidal volume (VT) does not fully reduce mortality. Lung-protective strategies should prioritize driving pressure (ΔP) over fixed tidal volume, as ultra-protective ventilation has not been shown to be superior, though larger clinical trials are needed for confirmation. Other studies suggest that the association between driving pressure and mortality may depend on patient age [150] or time since ARDS onset [151], underscoring the need for individualized support during lung-protective ventilation. Personalized ΔP and PEEP settings can be guided by lung imaging (e.g., electrical impedance tomography [152–155]), transpulmonary pressure measurements [156], and pleural pressure estimation. These approaches enable construction of dose–response curves for VT and ΔP and facilitate development of tailored lung-protective ventilation strategies [157] to achieve more uniform regional lung mechanics and reduce VILI. Both the ATS and ESICM guidelines advise against prolonged high-PEEP recruitment maneuvers [6, 7]. Additionally, ESICM [6] specifically recommends against brief high-pressure recruitment maneuvers.

#### Fraction of inspired oxygen (FiO_2_)

Oxygen therapy is a cornerstone of ARDS management, yet optimal oxygen saturation targets remain undefined. Traditional liberal strategies aim to prevent tissue hypoxia and organ dysfunction [158], but excessive oxygen exposure carries risks of oxidative injury [159], prompting consideration of conservative alternatives. However, no conclusive evidence favors either approach [160–164]. The Liberal Oxygenation versus Conservative Oxygenation in Acute Respiratory Distress Syndrome (LOCO_2_) trial [158] found that conservative PaO_2_ targets (55–70 mmHg) provided no benefit and may increase mortality and mesenteric ischemia, whereas the Intensive Care Unit Randomized Trial Comparing Two Approaches to Oxygen Therapy (ICU-ROX) [165], Handling Oxygenation Targets in the ICU (HOT-ICU) [166], and UK Intensive Care Unit Randomized Trial Comparing Two Approaches to Oxygen Therapy (UK-ROX) [167] trials reported no significant mortality differences between conservative and liberal or conventional oxygen therapies. Notably, lower oxygen targets may benefit specific subgroups, including critically ill children [168] and patients with severe COVID-19–related hypoxemia [169], underscoring that optimal targets depend on individual patient characteristics [170–172]. This is further supported by individualized treatment effect (ITE) models: the Pragmatic Investigation of Optimal Oxygen Targets (PILOT) trial [172] demonstrated that the predicted effect of lower versus higher SpO_2_ targets on 28-day mortality ranged from a 27% reduction to a 34% increase, with external validation favoring a shift from population-average to personalized targets [173]. Despite limitations, including risks of model overfitting, infeasibility of directly observing individual treatment responses, and dependence on sample size and variable selection, stratified and individualized oxygenation strategies hold promise for ARDS patients, though further prospective studies are needed to validate these models and support clinical translation.

#### Non‑invasive respiratory supports

Non-invasive respiratory support may be used initially in patients with ARDS. For non-intubated patients with ARDS or AHRF, the ESICM [6] recommends HFNO over conventional oxygen therapy (COT) to reduce intubation risk, whereas the ATS guidelines do not address this population. HFNO has been widely adopted since the FLORALI trial [174] and during the COVID-19 pandemic [26], owing to its good tolerability, ability to deliver PEEP of 3–5 cm H₂O, and reduction of anatomical dead space; consequently, patients managed with HFNO are now included in the updated global definition of ARDS [1]. However, outcomes with HFNO in immunocompromised patients with hypoxemic respiratory failure vary widely and depend on factors such as the type of immunosuppression and underlying etiology [175–177], underscoring the need for improved evidence-based risk stratification and individualized treatment strategies to optimize outcomes in this subgroup. Additionally, ESICM guidelines [6] recommend continuous positive airway pressure (CPAP) over COT and suggest considering CPAP or non-invasive ventilation (NIV) instead of HFNO to further reduce intubation rates in patients with COVID-19–related AHRF. Future research should focus on defining the optimal timing, duration, and modes of non-invasive and invasive mechanical ventilation [178] to clarify the therapeutic role of respiratory support in ARDS.

#### Veno-Venous Extracorporeal Membrane Oxygenation (VV-ECMO)

Both the ATS and ESICM guidelines recommend the use of Veno-Venous Extracorporeal Membrane Oxygenation (VV-ECMO) in severe ARDS [6, 7]. A meta-analysis [179] of the ECMO to Rescue Lung Injury in Severe ARDS (EOLIA) [180] and conventional ventilatory support versus extracorporeal membrane oxygenation for severe adult respiratory failure (CESAR) [181] trials suggests that VV-ECMO may reduce mortality in ARDS and may increase ventilator-, vasopressor-, and renal replacement therapy-free days, but it may also increase the risk of hemorrhage. However, we believe that evidence-based prediction should not be employed in isolation to define ECMO applicability, as research findings only reflect the prognosis of patients who eventually require intubation. In fact, VV-ECMO implementation requires a case-by-case approach. Firstly, before implementing VV-ECMO, simpler, non-invasive, cost-effective, and readily available interventions for ARDS (such as lung-protective ventilation and prone positioning) should be thoroughly explored and proven ineffective before escalating to more advanced treatments. Secondly, given that VV-ECMO is expensive, resource-intensive, highly invasive [182], and associated with serious complications, prognostic factors (including patient, disease, and center factors) should be carefully considered before intubation to maximize risk stratification. According to the results of EuroECMO-COVID [183], ECMO-SURGES [184], age-related ECMO studies [185, 186], and meta-analyses [187], we summarize the factors that may reduce ECMO mortality as follows: patient factors, such as younger age; pre-intubation disease factors, such as shorter duration of invasive mechanical ventilation, lower partial pressure of arterial carbon dioxide, and fewer comorbidities; and center factors, such as being treated at a center with extensive prior experience in using ECMO, where a higher number of cases is associated with better outcomes. Furthermore, optimizing the extracorporeal support environment during VV-ECMO implementation, including parameters such as blood flow rate [188], may provide safer and more effective support. Taken together, exploring suitable populations for ECMO and optimizing the technical implementation are the core paths to enhancing the value of ECMO and achieving precise support.

#### Prone positioning

Prone positioning is well established as beneficial for patients with ARDS. Physiological studies show that the prone position improves overall ventilation/perfusion matching by distributing the gas-to-tissue ratio more evenly along the dependent–nondependent axis and simultaneously reduces lung stress and strain [189, 190]. The PROSEVA trial [147] further supports this benefit: early application of prolonged (at least 16 h) prone positioning significantly reduced 28- and 90-day mortality by 16.8% and 17.4%, respectively, in critically ill ARDS patients with a PaO₂/FiO₂ ratio < 150 mm Hg. Consequently, both the ATS and ESICM guidelines recommend prone positioning for moderate-to-severe ARDS [6, 7]. However, clinical practice has shown heterogeneous results; an analysis in the ESICM guidelines [6] found no difference between prone and supine positioning in short- or long-term mortality, suggesting that the short-term physiological benefits may not always translate into long-term survival gains.

Heterogeneity is also evident in the effectiveness of prone positioning for ARDS patients receiving VV-ECMO. The Prone Positioning During Extracorporeal Membrane Oxygenation in Patients With Severe ARDS (PRONECMO) trial [190] showed that prone positioning did not reduce time to successful ECMO weaning, corroborating the earlier NCT03918603 study [191], which reported a trend toward higher 60-day mortality in patients with ARDS on VV-ECMO combined with prone positioning under ultra-lung-protective ventilation. These findings contrast with several studies and meta-analyses suggesting that prone positioning during ECMO can improve survival [192], particularly early survival [193, 194]. This heterogeneity may stem from several factors. First, 94% of ARDS patients in the PRONECMO trial had COVID-19 pneumonia, which may involve unique pathophysiological changes [190]; prolonged ECMO duration and its associated mortality [184, 190, 195, 196] could diminish any potential benefit of interventions applied early during ECMO support [197], raising uncertainty about whether these results apply to severe non–COVID-19-related ARDS. Second, the prevalence of prone positioning before ECMO initiation influences outcomes: in the PRONECMO trial [190], 95% of ECMO recipients had received prone positioning before ECMO, compared with 18%–85% in observational studies [192] (59% in the PRESERVE cohort [198]); pre-ECMO prone positioning was independently associated with improved survival [199], potentially offsetting additional benefits from prone positioning during ECMO. Third, the PRONECMO trial uniformly applied prone positioning without considering individual patient characteristics, such as lung recruitability or risk of overdistension, that might determine who benefits during ECMO [200]. Finally, in patients with low respiratory compliance where ultra-protective ventilation has already mitigated lung injury, the protective effect of prone positioning via tidal volume redistribution may be diminished [197, 201]. Thus, current evidence is insufficient to support routine use of prone positioning during ECMO, and further research is needed to assess lung lesion distribution in these patients.

Additionally, maintaining an awake prone position is an important supportive measure for non-intubated AHRF patients. The ESICM guidelines [6] specifically recommend awake prone positioning in non-intubated patients with COVID-19–related AHRF to reduce intubation, though optimal physiological parameters to monitor after treatment remain unclear. Assessing variables such as early improvement in the respiratory rate–oxygenation (ROX) index may help predict the risk of invasive mechanical ventilation or treatment failure [202]. However, as all enrolled patients in these studies were COVID-19 patients, future research should validate these findings in AHRF due to other etiologies. Therefore, the clinical value of awake prone positioning depends not only on the intervention itself, but also on the accurate identification and application of efficacy assessment indicators.

#### Fluid management

A conservative fluid strategy has been shown to improve clinical outcomes in ARDS. The FACTT study showed [149] that although 60-day mortality (the primary endpoint) did not differ between groups, patients assigned to conservative fluid management had shorter durations of mechanical ventilation and intensive care unit stay. However, when adopting this approach, clinicians must consider multiple variables, determine the patient’s fluid phase accurately, assess renal function [203], and monitor for potential adverse effects of fluid restriction. A post-hoc analysis of the HEMOPRED study [204] indicated that dynamic indicators of fluid responsiveness are helpful for individualizing fluid management in ARDS. Further research is needed to identify clinical markers that distinguish between the ebb and flow phases of ARDS to provide more evidence-based guidance for fluid therapy.

#### Neuromuscular Blocking Agents (NMBAs)

The role of neuromuscular blocking agents (NMBAs) in ARDS remains controversial, and any potential benefit likely depends on patient stratification, timing of administration, and the overall treatment strategy. NMBAs may benefit patients with ARDS by reducing VILI through improved ventilator synchrony, as well as by attenuating inflammation, decreasing alveolar fluid accumulation, and lowering systemic oxygen consumption [205, 206]. However, key clinical trials have yielded conflicting results: the ACURASYS trial [207] found that continuous cisatracurium infusion improved outcomes in patients with moderate-to-severe ARDS, whereas the ROSE trial [208] showed no such benefit. These discrepancies may stem from differences in study design, including ARDS definitions, timing of enrollment, sedation targets, ventilator strategies, and use of prone positioning, leaving no current consensus on NMBA management guidelines. The ATS [7] recommends early NMBA use (within 48 h of ARDS onset) in patients with severe ARDS (PaO₂/FiO₂ ≤ 100 mmHg), while the ESICM [6] advises against routine use in non-COVID-19 moderate-to-severe ARDS. Despite the lack of consensus, phenotypic stratification analyses accounting for population heterogeneity offer insights for precision application. Post hoc analyses [205, 209] suggest that younger patients (< 60 years) without life-limiting comorbidities, as well as those with higher respiratory system elastance (Ers), may derive greater benefit from NMBA therapy. Future studies should therefore integrate mechanistic research with data modeling to identify ARDS subphenotypes most likely to respond to NMBAs, thereby advancing personalized treatment strategies.

### Treatment of the underlying cause

Currently, no effective pharmacologic therapies are routinely used for ARDS. Therefore, prompt and accurate identification and treatment of potentially reversible causes may be critical. Infection, the most common cause of ARDS, is particularly challenging because causative microorganisms cannot be identified in 30%–60% of cases [2], which hampers etiologic management. Developing rapid pathogen identification technologies, such as metagenomic sequencing [210], could improve diagnostic capabilities and serve as an important strategy to enhance ARDS outcomes. However, overdiagnosis remains a concern, as positive results may reflect colonization rather than true infection. At the same time, while individualized treatment targeting specific etiologies [35] is a promising direction, it is equally important to determine whether distinct etiologies share common, clinically relevant pathological features. This further underscores the need to shift from etiology-oriented approaches toward phenotype-based classification and novel targeted therapies.

### Corticosteroids

Corticosteroids are key agents for anti-inflammatory and immunomodulatory therapy in ARDS, but their clinical benefits are substantially influenced by patient heterogeneity and variations in treatment protocols. The controversy largely stems from differences across trials in corticosteroid type, dosage, timing of initiation, and patient characteristics, including time of diagnosis and enrollment, sex, immune status, and comorbidities such as septic shock [211]. A large pooled analysis of randomized controlled trials [212–217] indicated that corticosteroids may reduce mortality and shorten the duration of mechanical ventilation and hospital stay [7, 218]. The DEXA-ARDS trial [212] demonstrated that early dexamethasone administration (within 24 h of ARDS onset) in patients with moderate-to-severe ARDS reduced both mechanical ventilation duration and overall mortality, although the trial’s enrollment rate was low. Similarly, the Community-Acquired Pneumonia: Evaluation of Corticosteroids (CAPE COD) trial [219], which initiated hydrocortisone within 24 h of diagnosis, showed that patients with severe community-acquired pneumonia (some meeting ARDS criteria) had lower 28-day mortality than those receiving placebo. Based on these findings, the ATS [7] and the Society of Critical Care Medicine (SCCM) [218] conditionally recommend corticosteroids for adults with ARDS. However, the ESCAPe trial [220] found that low-dose methylprednisolone given 72–96 h after diagnosis did not significantly reduce 60-day all-cause mortality in patients with severe community-acquired pneumonia, highlighting that treatment timing is crucial, missing the optimal window may abolish therapeutic efficacy.

Regarding safety, corticosteroids can cause adverse effects including hyperglycemia [221], hypernatremia [222], secondary infections [223, 224], gastrointestinal bleeding [225], and muscle weakness [222], all of which require close monitoring. Ongoing large-scale clinical trials (ISRCTN15076735, NCT05440851) and updated machine learning–based simulations [226] are expected to provide deeper and broader insights into the efficacy of corticosteroids in ARDS.

### Enrichment strategies to guide precision medicine in ARDS

#### Inflammopathic phenotype

Although current therapeutic approaches remain inconsistent and yield conflicting evidence, understanding of inflammation and immune dysregulation in ARDS continues to evolve, shifting from a static, symptom-centered view toward a dynamic, mechanism-based perspective. The host immune response in ARDS is characterized by complexity, heterogeneity, compartmentalization, and temporal dynamics [63]. Consequently, treatment may require moving beyond simplistic, high-failure-rate “one-size-fits-all” strategies (and away from the anti- versus pro-inflammatory dichotomy) toward enrichment approaches that incorporate individual patient characteristics, such as biomarkers and phenotypic classification. For example, glucocorticoid efficacy in COVID-19 patients appears to interact with inflammatory phenotypes [126], supporting the potential of predictive enrichment. A secondary analysis of the HARP-2 trial found that simvastatin improved survival in a hyperinflammatory ARDS subgroup [135], whereas studies of rosuvastatin showed no benefit [227]. This heterogeneity underscores the need to stratify ARDS patients into hyperinflammatory and hypoinflammatory subgroups.

Interleukin-6 (IL-6) antagonists, such as tocilizumab, are associated with reduced 28-day all-cause mortality in hospitalized patients with COVID-19, particularly when combined with corticosteroids, which enhances anti-inflammatory effects [228]. However, the included studies were not restricted to ARDS patients. Data suggest that individuals with higher circulating IL-6 concentrations may derive greater benefit from tocilizumab [229].

Similarly, blockade of the IL-1 signaling pathway may be effective primarily in ARDS patients with specific hyperinflammatory phenotypes. Extensive immune activation can lead to macrophage activation–like syndrome (MALS), characterized by hyperferritinemia, hepatobiliary dysfunction, hypertriglyceridemia, and disseminated intravascular coagulation [63, 230]. The IL-1 receptor antagonist (IL-1RA) anakinra has been shown to improve 7-day outcomes in septic patients with MALS [231]. Secondary analyses of the HARP-2 [232] and ROSE [208] trials further linked hyperferritinemia to poor prognosis in ARDS [233], leading to the hypothesis that ARDS patients with MALS, defined by elevated serum ferritin, may benefit from IL-1 blockade [2]. However, this cannot be extrapolated to all ARDS cases. Notably, some patients who develop ARDS early in COVID-19 exhibit lower inflammatory markers (e.g., ferritin) than those with other cytokine storm syndromes [234], highlighting the necessity of biologically informed intervention. For instance, the SAVE-MORE trial [235] demonstrated that early anakinra administration guided by plasma soluble urokinase plasminogen activator receptor levels reduced 28-day mortality in moderate-to-severe COVID-19. Additional evidence suggests anakinra may protect certain ARDS patients [236], particularly those with baseline lymphocyte dysfunction [237], and the ImmunoSep trial [238] supports the feasibility of biomarker-guided anakinra use. Overall, the utility of IL-1RAs in ARDS depends on predictive enrichment through biomarker-driven patient selection.

Other immune-related targets, such as the IL-8/CXCR1/2 axis [66], have shown preclinical promise but yielded disappointing clinical results. A phase III trial (NCT04878055) [239] of reparixin, an IL-8 pathway inhibitor, failed to meet its primary endpoint: the proportion of patients alive and free of respiratory failure at day 28 did not differ significantly from placebo. Potential reasons include suboptimal timing, route, or duration of inhibitor administration. IL-8 is highly compartmentalized; in ARDS patients, the bronchoalveolar lavage fluid (BALF)-to-serum IL-8 ratio is approximately 20-fold higher than in controls [97]. Alveolar inflammation correlates positively with ARDS mortality [95], and early inhibitor administration may mitigate persistent alveolar inflammation. In later disease stages, however, disruption of the alveolar-capillary barrier may allow local inflammation to become systemic, reducing the chemokine gradient, a change negatively correlated with mortality, and thereby diminishing drug efficacy [66]. Finally, acetaminophen, which targets CFH to reduce ARDS incidence in sepsis, did not significantly improve days alive and free of organ support in critically ill patients in the ASTER trial [64]. Larger studies are therefore needed to explore heterogeneity in acetaminophen treatment response.

#### Coagulopathic phenotype

Coagulopathy plays a key role in the pathogenesis of ARDS, and classifying patients by coagulopathic phenotype may help guide therapy. Cluster analysis of data from an ARDS cohort showed that recombinant human thrombomodulin (rhTM) was effective in patients with severe coagulopathy [131]. In critically ill patients with COVID-19, a strategy of full-dose versus standard-dose prophylactic anticoagulation (heparin or low-molecular-weight heparin) reduced thrombotic complications but increased bleeding risk, with no significant increase in mortality [240]. These findings indicate that the coagulopathic phenotype represents a clinically meaningful ARDS subclass, as reflected by differential responses to coagulation-targeted interventions and the risk–benefit profile of anticoagulant strategies. Stratifying ARDS patients by coagulation status may therefore enable individualized treatment that balances efficacy and safety while addressing the central role of coagulopathy in disease progression.

#### Other agents

Additional molecular targets implicated in ARDS include receptor-interacting protein kinase 3 (RIPK3) [241], the NLRP3 inflammasome [242], E2F transcription factor 2 (E2F2) [243], and heme oxygenase-1 (HMOX1) [244]. Therapeutic strategies targeting these molecules with specific inhibitors or modulators hold translational promise and may provide novel, mechanism-based approaches to improve outcomes in ARDS.

### Cell-targeted therapy

#### Mesenchymal Stromal Cells (MSCs)

Novel cell-based therapies, including mesenchymal stromal cells (MSCs) and extracellular vesicles [245], hold promise for ARDS treatment. MSCs act through multiple mechanisms: they exert anti-inflammatory and immunomodulatory effects [246], promote tissue repair, reduce pulmonary edema, and enhance pathogen clearance [2, 22, 247, 248]. Specifically, MSCs alleviate ALI by upregulating cluster of differentiation 24 expression, shifting neutrophils toward an anti-inflammatory phenotype, and suppressing ROS and oxidative stress [249]. MSCs engineered to express prostaglandin E₂ (PGE₂) [250] further drive macrophage polarization from the pro-inflammatory M1 to the anti-inflammatory M2 phenotype, characterized by decreased levels of interferon-γ (IFN-γ), tumor necrosis factor-α (TNF-α), interleukin-6 (IL-6), and IL-1β, along with increased production of the anti-inflammatory cytokine IL-10. MSCs may also dampen excessive inflammation by inhibiting T-cell [251] and NK cell proliferation [247].

Clinical evidence suggests that MSCs are generally safe and may reduce mortality in ARDS [252] (Table 2). However, results from key trials have been markedly heterogeneous. The Repair of Acute Respiratory Distress Syndrome in COVID-19 by Stromal Cells (REALIST-COVID) trial [253] found that a single intravenous dose of 400 × 10⁶ ORBCEL-C cells (CD362-enriched, umbilical cord–derived MSCs) did not improve primary or secondary outcomes related to pulmonary organ dysfunction in patients with moderate-to-severe COVID-19–associated ARDS when administered within 72 h of symptom onset. Although ORBCEL-C modulated the peripheral blood transcriptome, this effect did not translate into clinical benefit. In contrast, earlier findings from the STAT program appeared more encouraging: a preliminary study (NCT02097641) [254] reported that MSCs attenuated lung injury in ARDS, particularly by reducing angiopoietin-2 (Ang-2), IL-6, and TNF receptor-1 concentrations in BALF. However, the subsequent larger STAT trial (NCT03818854) [255] yielded negative results, showing no significant difference in the primary endpoint (oxygenation index at 36 h post-administration) or most secondary outcomes. This discrepancy underscores the challenges in translating preclinical findings into clinical efficacy.

**Table 2:** Clinical trials of MSC interventions for ARDS

Clinical Trials	Study phase	Patient population	Case	MSC source	Delivery time	Dose	Route	Principal findings	Concomitant corticosteroid therapy	Ref
NCT02097641	2a	Moderate-to-severe ARDS	17	BM-MSCs	After clinically stable for 2 h	10 × 10^6^/kg PBW at a single infusion	i.v	MSC treatment significantly reduced airspace total protein, Ang-2, IL-6, and sTNFR-1 concentrations	NA	[254]
NCT03818854	2b	Moderate-to-severe ARDS	59	BM-MSCs	After reaching a period of baseline stability	10 × 10^6^/kg PBW at a single infusion	i.v	There was no difference in the primary outcome measure, the oxygenation index. However, plasma protein biomarkers and gene expression analysis identified subgroups of patients with different treatment responses	Yes	[255]
NCT03042143	2	Moderate-to-severe COVID-19 ARDS	30	UC-MSCs	Within 72 h of the onset of moderate-to-severe ARDS caused by COVID-19	400 × 10^6^ ORBCEL-C MSCs at a single infusion	i.v	A single dose of 400 × 10⁶ ORBCEL-C MSCs was safe and well-tolerated. There were no significant differences in the primary safety outcomes (incidence of serious adverse events and oxygenation index at day 7)	Yes	[253]
NCT04371393	2/3	Moderate-to-severe COVID-19 ARDS	112	BM-MSCs	Patients received two infusions of the study product during the first week, with the second infusion 4 days after the first infusion (±1 d)	2 × 10^6^ cells/kg at two infusions	i.v	Although MSCs are safe, they did not improve 30-day survival rates or 60-day ventilator-free days in patients with moderate-to-severe COVID-19-related ARDS	Yes	[260]
NCT04615429	NA	Moderate-to-severe COVID-19 ARDS	10	BM-MSCs	Within 96 h of ARDS onset, and (if applicable) within 72 h of orotracheal intubation	1 × 10^6^ cells/kg at a single infusion	i.v	MSCs treatment did not significantly improve the PaO_2_/FiO_2_ ratio on day 7, but it is safe and might accelerate patients' clinical recovery and discharge	Yes	[262]
NCT04333368	2b	Moderate-to-severe COVID-19 ARDS	21	UC-MSCs	Within 96 h of ARDS onset	3 × 10^6^/kg given in 3 intravenous injections at 48-h intervals	i.v	MSC is safety and no adverse reactions were observed within 1 year. The persistence of lung opacities for 1 year, accompanied by impaired diffusion capacity of the lung for carbon monoxide, does not appear to lead to more severe functional impairment than that observed 1 year after ARDS caused by non-COVID-19	No	[263]
IRCT20130812014333N164	NA	COVID-19 ARDS	21	Placenta	NA	1.5–2 × 10^9^ EVs/kg, administered for two consecutive days	i.v	hPMSC-sEVs treatment was safe and effective, and could significantly reduce patient mortality	NA	[264]
IRCT20200217046526N2	2	COVID-19 ARDS	19	Perinatal tissues MSC-derived EVs	ICU admission < 48 h	Two consecutive injections of MSCs (1 × 10^8^ cells) or one dose of MSCs (1 × 10^8^ cells) followed by one dose of MSC-derived EVs	i.v	MSCs infusion was associated with reduced levels of inflammatory cytokines such as IL-6, TNF-α, IFN-γ, and CRP	Yes	[265]
NCT03807804	2	ARDS caused by pneumonia	20	BM-MSCs	Within 72 h of ARDS diagnosis	9.0 × 10^8^ cells of invimestrocel at a single infusion	i.v	Invimestrocel was well-tolerated, and when combined with standard treatment, it did not significantly increase ventilator-free days but may improve survival	NA	[266]
NCT04493242	2	Moderate-to-severe COVID-19 ARDS	68	BM-MSC-EVs	Time from the First COVID-19 Diagnosis to First ExoFlo Dose Date (days), Mean (SD) ExoFlo 15 mL: 10 (6.55); ExoFlo 10 mL: 9.1 (4.36)	10 mL or 15 mL of ExoFlo™ on day 1 and day 4	i.v	ExoFlo (15 mL dose) is safe in patients with severe or critical COVID-19 respiratory failure. In participants aged 18 to 65, the risk reduction in 60-day mortality was improved	Yes	[267]

*MSCs* mesenchymal stromal cells, *ARDS* acute respiratory distress syndrome, *PBW* predicted body weight, *i.v.* intravenous, *Ang-2* angiopoietin-2, *IL-6* interleukin-6, *sTNFR-1* soluble tumor necrosis factor receptor-1, *UC* umbilical cord, *BM* bone marrow, *TNF-α* tumor necrosis factor-α, *IFN-γ* interferon-γ, *ICU* intensive care unit, *CRP* C-reactive protein, *EVs* extracellular vesicles, *hPMSC-sEVs* human placental mesenchymal stromal cell–derived small extracellular vesicles, *PaO₂* partial pressure of arterial oxygen, *FiO₂* fraction of inspired oxygen, *NA* not applicable

Several factors hinder the clinical translation of MSC therapy. Patient heterogeneity, suboptimal dosing regimens, and dependence on the local pulmonary microenvironment all contribute to variable outcomes. The STAT trial [255] noted that baseline biomarker profiles in COVID-19–related ARDS differ from those in classical ARDS, and characteristics such as sex may influence treatment response [252, 256]. Additionally, variations in MSC source, manufacturing, storage, timing, dose, frequency, and route of administration likely affect efficacy. Notably, the bone marrow-derived, cryopreserved, single-dose intravenous regimen used in the STAT trial [255] may not represent the optimal strategy. Moreover, the immunoregulatory function of MSCs is modulated by the local inflammatory milieu, a phenomenon termed “MSC licensing” [257, 258]. Concomitant therapies, particularly corticosteroids [259–261], may further alter MSC activity and potentially blunt their anti-inflammatory and antifibrotic effects. Although MSCs generally have a favorable safety profile, the REALIST-COVID trial [253] reported three malignancies diagnosed during 2-year follow-up in the MSC-treated group, highlighting the need for long-term safety monitoring.

Given the limited sample sizes of existing studies, future efforts should prioritize large-scale, high-quality, and standardized clinical trials embedded within a precision medicine framework. Integrating phenotypic stratification with dynamic assessment of the pulmonary microenvironment will be essential to identify ARDS subpopulations most likely to benefit from MSC therapy, define its appropriate clinical indications, and establish its true therapeutic value.

#### Other cell-targeted therapies for ARDS

**Fig. 5:**
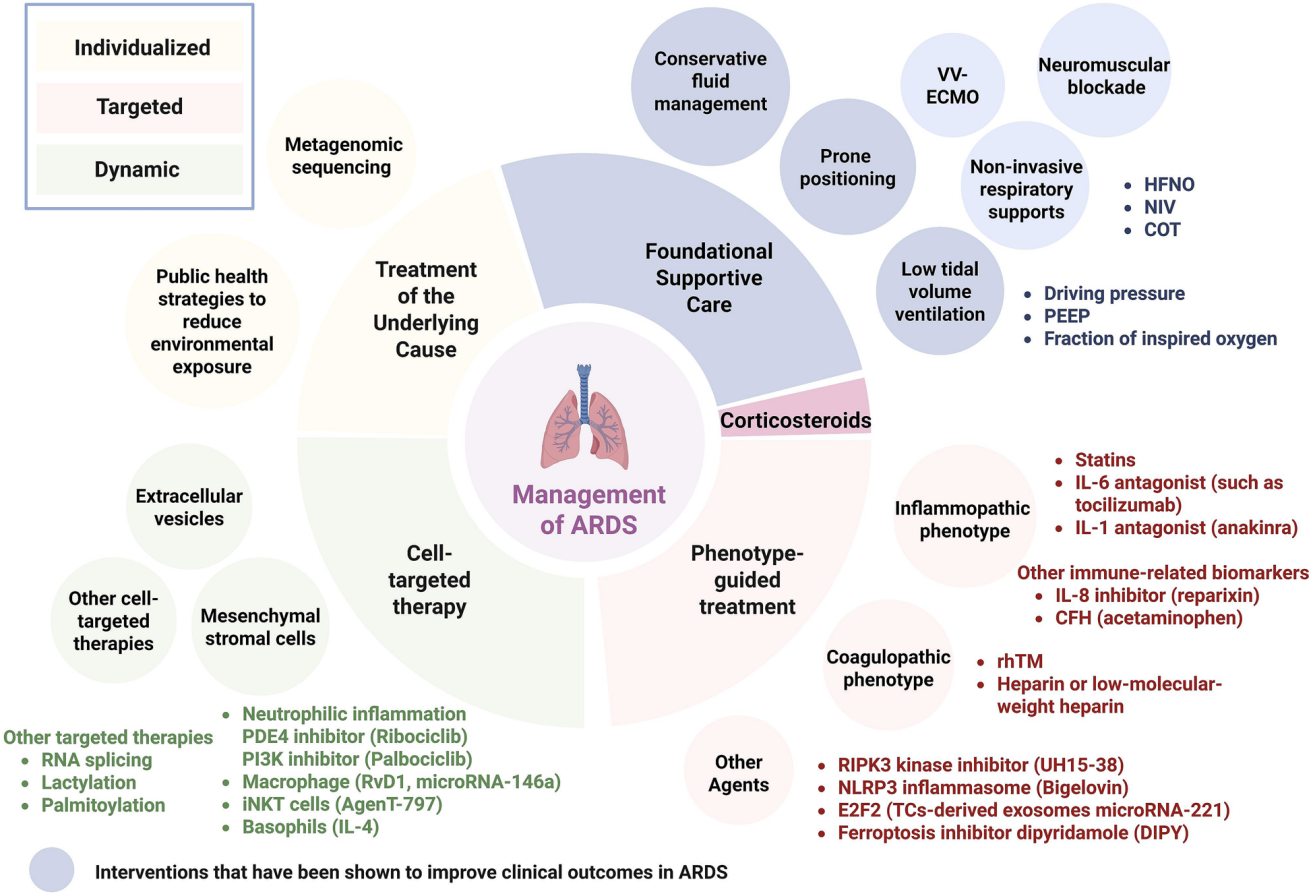
Potential treatments for acute respiratory distress syndrome (ARDS). Advances in research have expanded the therapeutic landscape for ARDS beyond foundational supportive care and management of underlying causes, now encompassing phenotype-guided and cell-targeted strategies. Given the clinical and biological heterogeneity of ARDS, growing evidence suggests that implementing individualized, targeted, and dynamic precision medicine will be a challenging yet exciting endeavor over the next decade. VV-ECMO: Veno-venous extracorporeal membrane oxygenation; HFNO: High-flow nasal oxygen; NIV: Non-invasive ventilation; COT: Conventional oxygen therapy; PEEP: Positive end-expiratory pressure; IL-1: Interleukin-1; IL-4: Interleukin-4; IL-6: Interleukin-6; IL-8: Interleukin-8; CFH: Cell-free hemoglobin; rhTM: Recombinant human thrombomodulin; RIPK3: Receptor-interacting protein kinase 3; NLRP3: NOD-, LRR- and pyrin domain-containing protein 3; E2F2: E2F transcription factor 2; PDE4: Phosphodiesterase 4; TCs-derived exosomes microRNA-221: Telocyte-derived exosomal microRNA-221; PI3K: Phosphoinositide 3-kinase; iNKT: Invariant natural killer T

Cell-targeted therapies are expected to be potential treatments for ARDS. Neutrophils [268, 269], macrophages [270, 271], invariant natural killer T (iNKT) cells [272], and basophils [273] are common targets. In addition to direct cell targeting, ARDS can also be intervened and regulated through approaches such as RNA splicing [274], lactylation [275], and palmitoylation [276]. However, it is worth noting that although these therapies (Table 3) show promising potential in the treatment of ARDS, most are still in the preclinical stage and lack in-depth research on their effectiveness, appropriate treatment regimens (including optimal dosage and timing), and safety in ARDS patients. Studies have shown that basophils primarily reduce inflammation in ARDS model mice by producing interleukin-4 (IL-4), which acts on neutrophils [273]. However, establishing a causal relationship between basophil counts and the pathogenesis of ARDS in humans remains challenging. More data are needed to clarify its clinical translational value. Figure 5 summarizes the potential treatments for ARDS.

**Table 3:** Molecular- and cell-targeted therapies for ARDS

Targeted objective	Agent(s)	Model	Mechanism	Key effects	Ref
PDE4	Ribociclib	ARDS: in vivo mouse (LPS)	By targeting the neutrophilic cAMP-PKA signaling pathway, it can be used to treat neutrophil-mediated ARDS	↓ROS,↓Chemotactic responses (integrin levels and adhesion)	[268]
PI3K	Palbociclib	ARDS: in vivo mouse (LPS)	By targeting PI3K activity, neutrophil inflammation can be inhibited, including superoxide anion generation, ROS formation, elastase degranulation, and chemotactic responses	↓The distortion of pulmonary architecture,↓Pulmonary interstitial oedema,↓Interalveolar septal thickening,↓Haemorrhagic conditions,↓Infiltration of Ly6G-positive cells,↓p-AKT	[277]
TRAM	RvD1	ARDS: in vivo mouse (LPS and/or Escherichia coli) ARDS: in vitro mouse TRAMs (LPS)	Improves TRAM self-renewal and phagocytosis via the ALX/MAPK14/S100A8/A9 signaling pathway	↓Neutrophil infiltration,↓Pulmonary pathological changes,↓ALI scores,↑CD206,↑Arg1,↓CD86,↓iNOS	[270]
AMs	MLNPs for delivery of microRNA-146a	ARDS: in vitro oscillatory pressure model of barotrauma ARDS: in vivo mouse (hemorrhagic shock)	Targeting AMs, increasing miR-146a levels in AMs, reducing the expression of TRAF6 and the production of macrophage-specific cytokine/chemokine	↓MIP-1α,↓IL-8,↓TRAF6 levels,↓IL-6	[271]
iNKT cells	AgenT-797	21 patients who required mechanical ventilation (5 received VV-ECMO)	It rescues exhausted T cells and rapidly activates both innate and adaptive immunity (activating dendritic cells and preferentially killing M_2_ macrophages)	↑IL-1RA,↑IL-4,↓IL-7,↓TNF-α,↓IL-1β,↓IL-10	[272]
Basophils	IL-4	ARDS: in vivo mouse (LPS) ARDS: in vitro mouse BMDMs and human THP-1 cells (LPS)	By producing IL-4, which acts on neutrophils, it inhibits the expression of anti-apoptotic genes and alleviates lung inflammation	↓Production of pro-inflammatory mediators (*Il1a*, *Il1b*, and *Cxcl2*),↓Anti-apoptotic genes expression (*Bcl2a1a, Bcl2a1b*, and *Bcl2a1d*)	[273]
RIPK3	UH15-38	Lung injury: in vivo mouse (IAV)	By blocking necroptosis and preventing deleterious lung inflammation, without affecting viral clearance in severe influenza	↓Levels of inflammatory cytokines and neutrophil chemoattractants (e.g., IL-1β, IL-6, IL-18, TNF-α)	[241]
NLRP3 inflammasome	Bigelovin	ARDS: in vivo mouse (LPS) ARDS: in vitro mouse BMDMs and human THP-1 cells (LPS)	It is possible to inhibit RACK1 by directly binding to its Cys168 site, thereby preventing NLRP3 oligomerization (active conformation) and improving ARDS	↓NF-κB activation,↓IL-1β,↓Caspase-1	[242]
E2F2	TCs-derived exosomes microRNA-221	ARDS: in vivo mouse (LPS)	Promoting angiogenesis and alleviating ARDS via the JAK-STAT-miR-221-E2F2 axis	1. Promoting angiogenesis in MVECs under LPS-induced inflammation. 2.↓TNF-α,↓IL-6,↓Pulmonary edema	[243]
Ferroptosis	DIPY	ARDS: in vivo mouse (LPS) Ferroptosis: hAOs (RSL3) 5 patients with moderate-to-severe ARDS	By downregulating HMOX1, ferroptosis and lung injury were effectively alleviated in mouse models and hAOs	↓Lung wet-dry ratio and total protein in BALF,↓Total cell counts,↓Neutrophil infiltration,↓Inflammatory factors (TNF-α, and IL-1β)	[244]

*ARDS* acute respiratory distress syndrome, *ALI* acute lung injury, *AMs* alveolar macrophages, *IL-1RA* interleukin-1 receptor antagonist, *IL-1β* interleukin-1β, *IL-4* interleukin-4, *IL-6* interleukin-6, *IL-7* interleukin-7, *IL-8* interleukin-8, *IL-10* interleukin-10, *IL-18* interleukin-18, *TNF-α* tumor necrosis factor-α, *PDE4* phosphodiesterase 4, *LPS* lipopolysaccharide, *cAMP* cyclic adenosine monophosphate, *PKA* protein kinase A, *ROS* reactive oxygen species, *PI3K* phosphoinositide 3-kinase, *p-AKT* phosphorylated AKT, *RvD1* resolvin D1, *TRAMs* tissue-resident alveolar macrophages, *ALX* lipoxin A4 receptor, *MAPK* mitogen-activated protein kinase, *iNOS* inducible nitric oxide synthase, *MLNPs* mannosylated lipid nanoparticles, *TRAF6* TNF receptor–associated factor 6, *iNKT cells* invariant natural killer T cells, *VV-ECMO* veno-venous extracorporeal membrane oxygenation, *IAV* influenza A virus, *RACK1* receptor for activated C kinase 1, *TCs-derived exosomes microRNA-221* telocyte-derived exosomal microRNA-221, *E2F2* E2F transcription factor 2, *NLRP3* NOD-, LRR- and pyrin domain–containing protein 3, *MVECs* murine vascular endothelial cells, *DIPY* dipyridamole, *hAOs* human airway organoids, *RSL3* RAS-selective lethal 3, *HMOX1* heme oxygenase-1, *BALF* bronchoalveolar lavage fluid

## Clinical translation and long-term management

With the rapid development of precision medicine in ARDS, the key challenge is no longer limited to identifying biologically distinct phenotypes, but also involves translating these insights into clinically meaningful therapeutic strategies. This section focuses on the translational barriers that remain, including the limitations of conventional clinical trial designs, the need for phenotype-based enrichment strategies, and the importance of extending ARDS care beyond short-term survival toward multidimensional long-term support for survivors and their families.

### Phenotype-driven clinical trial design

#### Enrichment strategy

Clinical trial design is critical for translating promising interventions into meaningful clinical benefit, a process that is especially challenging given the high heterogeneity of ARDS. Conventional trial designs typically evaluate a single intervention against a single comparator in a relatively homogeneous study population, yielding results that reflect average treatment effects across the entire cohort. In contrast, ARDS exhibits marked clinical and biological heterogeneity, and reliance on average effects alone often obscures true therapeutic benefits in specific patient subgroups. Therefore, balancing prognostic enrichment (guided by outcomes such as mortality) and predictive enrichment (selecting patients most likely to respond to a specific therapy) is essential for precision treatment and experimental design in ARDS. For example, a secondary analysis of the ROSE trial indicated [142] that patients with a hyperinflammatory phenotype, and higher mortality, were overrepresented in the study, possibly due to its unique early recruitment and severity stratification [142]. This finding suggests that trials should adopt predictive enrichment criteria, enrolling only those patients most likely to benefit. Moving forward, integrating phenotypic identification and enrichment strategies into the initial trial design phase will be crucial for accurately capturing and rigorously validating the clinical benefits of precision therapy in ARDS.

#### Challenges and opportunities

Research on ARDS phenotyping is advancing from descriptive identification toward clinical translational validation. However, its successful implementation depends on parallel progress in the evidence base, diagnostic capacity, and research paradigms.

First, existing subphenotypes have been identified primarily through retrospective analyses. Prospective validation in clinical trials stratified by subphenotype is therefore needed to confirm the association between phenotype and heterogeneous treatment responses [70, 115]. Encouragingly, the ImmunoSep trial [238], described as firing the first shot in the precision medicine war, demonstrated that precision immunotherapy with anakinra in septic patients exhibiting MALS (defined by high ferritin) improved organ dysfunction by day 9. Second, the development of point-of-care testing technologies addresses key requirements for biomarker detection: timeliness, availability, stability, and reproducibility. Systems such as the FilmArray® platform [278] and the PHIND study (NCT04009330) now enable rapid, independent biomarker detection and real-time assignment of ARDS subphenotypes in clinical practice.

More specifically, achieving robust clinical interpretability for phenotyping requires deeper mechanistic studies to elucidate its biological underpinnings, alongside larger multicenter investigations to account for the inherent heterogeneity of clinical samples. Notably, the temporal dynamics of ARDS phenotypes underscore the need for iterative phenotyping to guide therapy [279], supporting a shift toward adaptive, longitudinal immunomodulatory interventions [280]. Finally, trajectory-based stratification can optimize experimental design. When the optimal biomarker threshold is initially uncertain, risk- or effect-based interim models can identify subgroups with differential treatment effects. This information can then inform stratified randomization and/or adaptive predictive enrichment, enabling real-time adjustments to the trial design. Thus, the future of ARDS phenotyping lies not merely in identifying additional subtypes, but more importantly in the integrated incorporation of dynamic phenotypes, biomarker assessment, and adaptive trial design. Such integration will ultimately transform precision therapy from a conceptual framework into a clinically testable and implementable reality.

#### Platform trial

Adaptive platform trials offer considerable promise as a more efficient framework for precision therapy research in ARDS, one better aligned with the syndrome’s inherent heterogeneity. Given the complexity of identifying ARDS subphenotypes, the limitations of traditional trial designs, and the resulting inefficiencies in drug development, adaptive platform trials may help overcome these barriers. These trials enable the simultaneous evaluation of multiple interventions within a single master protocol, facilitating patient stratification by clinical characteristics, biomarkers, and phenotypes, and allowing concurrent assessment of diverse therapeutic effects [63]. The PANTHER trial (Precision Medicine Adaptive Platform Network Trial in Hypoxemic Acute Respiratory Failure; https://panthertrial.org/) exemplifies this approach, accelerating pharmacological therapy development for ARDS by focusing on patients with hyperinflammatory and hypoinflammatory phenotypes [281]. By integrating flexibility, precise stratification, and efficient multi-intervention evaluation, platform trials mark a paradigm shift in ARDS research, from a historical focus on average treatment effects toward the identification of subgroup-specific benefits. In this context, adaptive platform trials not only address the longstanding bottleneck in translating promising therapies into effective clinical practice but also establish a methodological foundation for personalized treatment strategies tailored to specific patient subgroups.

### Multidimensional long-term support

ARDS research is undergoing an important paradigm shift from short-term survival to multidimensional long-term outcomes. Survivors often experience persistent physical, cognitive, and psychological sequelae, with some cohorts reporting functional decline up to five years after discharge. In fact, certain patients exhibit ongoing physical impairment even five years post-discharge. Notably, this study identified the midazolam- equivalent dose as an independent predictor of decline in at least one functional measure [282]. These findings indicate that acute-phase interventions, including analgesia, sedation, hemodynamic management, and respiratory support, exert a profound impact on long-term prognosis.

However, ARDS outcomes are not determined by acute-phase care alone. Adverse prognostic factors such as age [20, 150], pre-existing health status, and disease interactions [45] are equally important. For instance, long-term quality of life among VV-ECMO survivors varies considerably [183, 283–285], influenced by both follow-up duration and baseline disease severity.

Addressing these challenges necessitates a continuum-of-care perspective that supports patients and their families across all stages of ARDS through sustained, multidimensional functional assistance. First, healthcare professionals, patients, and families should receive comprehensive education about ARDS outcomes and the critical care continuum. Adherence to the evidence-based ABCDEF bundle [286], (A) Assess, prevent, and manage pain; (B) Both spontaneous awakening trials and spontaneous breathing trials; (C) Choice of analgesia and sedation; (D) Delirium: assess, prevent, and manage; (E) Early mobility and exercise; (F) Family engagement and empowerment, is essential to minimize iatrogenic harm, improve outcomes, and preserve patients’ sense of self-worth and dignity. Second, priority must be given to basic and translational research on risk stratification, the roles of nutrition and rehabilitation, and the delineation of prognostic trajectories to enhance continuity in care, nutrition, follow-up, rehabilitation, and recovery. Third, longitudinal, granular data on ARDS outcomes should be integrated, and consistent standards established for patient- and family-centered data collection. Fourth, multidisciplinary teams must communicate promptly and effectively with patients and families, delivering respectful, compassionate care and actively involving family members in end-of-life decision-making. Only through accurate identification of target populations, optimized implementation of technologies, and strengthened follow-up support can ARDS management truly evolve into a patient-centered, integrated care model.

## Conclusions

ARDS is a severe clinical syndrome characterized by high morbidity and mortality, underscoring the urgent need for effective therapies beyond current supportive care. Drawing on current understanding of ARDS epidemiology, etiology, pathophysiology, definition, and diagnosis, we have summarized the application of diverse therapeutic approaches in preclinical and clinical settings (Fig. 5). In addition to public health strategies aimed at reducing environmental exposures and lowering background ARDS risk, rapid pathogen identification through metagenomic sequencing now enables timely targeting of underlying causes.

**Fig. 6:**
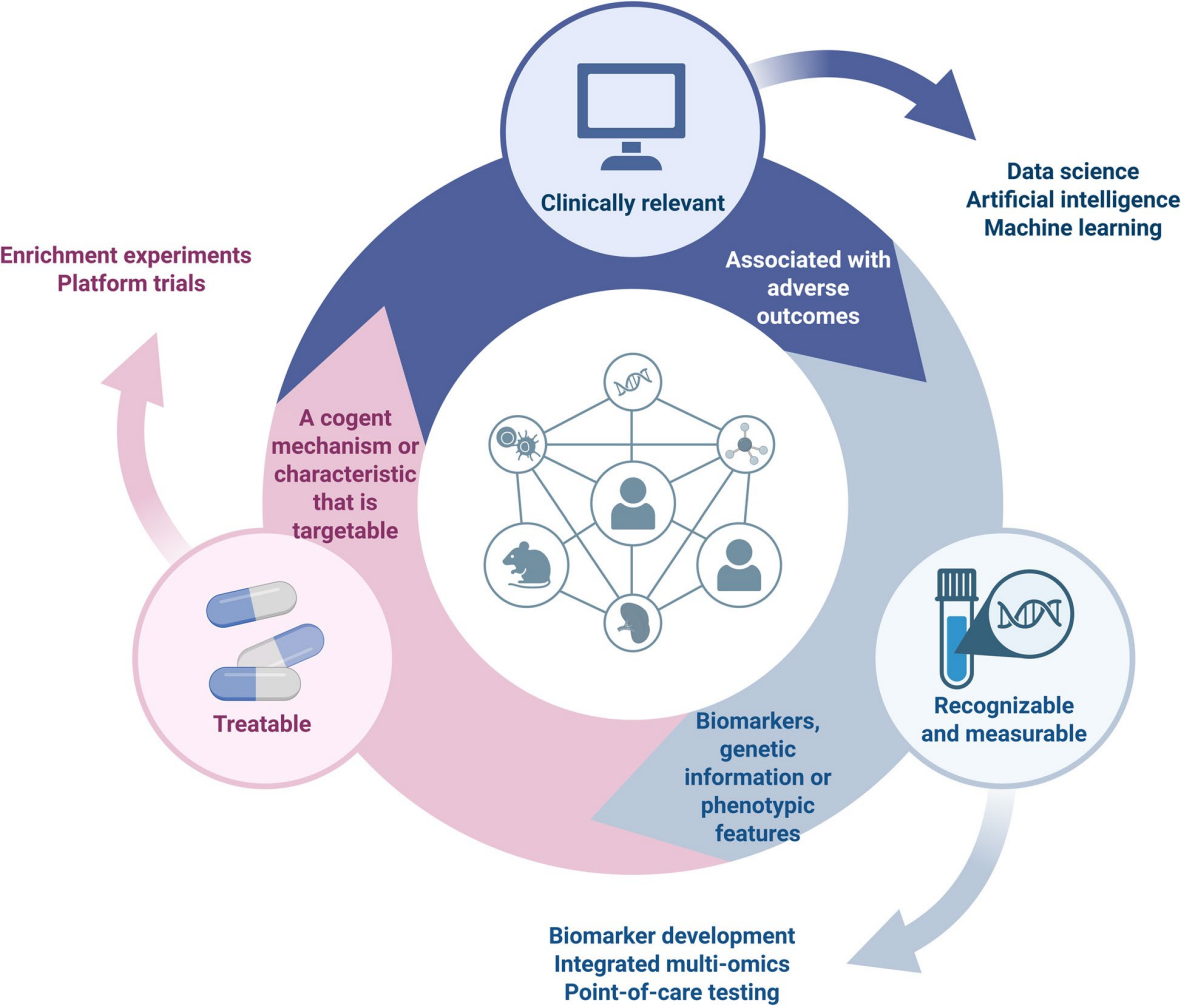
Treatable trait-oriented precision medicine framework for acute respiratory distress syndrome (ARDS). The figure illustrates an iterative framework for precision medicine in ARDS based on the identification and refinement of clinically actionable treatable traits. These traits should be clinically relevant, measurable through biomarkers or phenotypic features, and linked to targetable mechanisms or modifiable disease processes. Within this iterative framework, data science, artificial intelligence (AI) and machine learning can integrate multidimensional clinical and molecular datasets to identify candidate actionable traits, whereas biomarker development, integrated multi-omics and point-of-care testing can support their validation and dynamic stratification. Enrichment experiments and platform trials can then evaluate targeted interventions in biologically defined populations, with trial outputs feeding back to refine trait definitions, stratification criteria and therapeutic targets

However, it is critically important to recognize that ARDS exhibits marked clinical and biological heterogeneity, encompassing variations in clinical features, etiology, host response, injury mechanisms, and disease severity. This spatiotemporal heterogeneity highlights the necessity of precision medicine strategies focused on “treatable traits” to optimize patient outcomes (Fig. 6). Realizing this paradigm shift requires deeper insights into ARDS pathophysiology and the integration of multidimensional clinical, physiological, and molecular data to establish robust, translatable phenotyping frameworks. Building on this foundation, innovative trial designs, including predictive enrichment trials and adaptive platform trials, will be essential to effectively link phenotypic stratification with precision interventions. Collectively, these advances will accelerate the transition to individualized, targeted, and dynamically adjusted ARDS treatment, ultimately transforming the clinical management of this devastating syndrome.

Despite significant progress in ARDS research, numerous unresolved challenges continue to hinder the translation of scientific advances into improved clinical outcomes. Although the updated definition enhances clinical applicability and facilitates earlier detection, its impact on key epidemiological parameters, such as incidence, mortality, and long-term sequelae, remains unclear. Prospective studies are needed to validate its prognostic value, and further refinement through integration of respiratory mechanics, radiomics, and multi-omics biomarkers is essential to optimize personalized treatment guidance. Critical gaps also persist in both supportive and pharmacological care: consensus on individualized strategies, including lung-protective ventilation, optimal timing and mode of mechanical ventilation, and oxygen saturation targets, is lacking, as traditional “average-effect” interventions prove insufficient. Effective pharmacotherapies beyond supportive care remain scarce; the patient populations most likely to benefit from NMBAs and corticosteroids, along with their optimal regimens, are still undefined, and the clinical translation of novel targeted therapies such as MSCs requires definitive confirmation.

At a deeper level, these challenges reflect an incomplete understanding of ARDS pathophysiology. The syndrome’s marked heterogeneity demands foundational research integrating multi-omics technologies with humanized models and organoids to dissect molecular and cellular drivers, while artificial intelligence and machine learning are indispensable for analyzing high-dimensional imaging, physiological, and longitudinal clinical data to identify robust biomarkers, clinically relevant phenotypes, and actionable “treatable traits”. ARDS subtyping research now stands at a critical juncture, transitioning from identification to prospective validation and clinical application, with key questions surrounding subtype interrelationships, practical biomarker implementation, temporal stability, and whether subtype-directed therapies can improve outcomes in predictive enrichment or adaptive platform trials. Concurrently, the long-term, multidimensional burden on survivors, functional, cognitive, and psychological, remains poorly understood, necessitating systematic evaluation of these outcomes and integration of patient and family perspectives to prioritize quality-of-life–centered research beyond traditional mortality metrics. Overcoming these multifaceted challenges demands a collaborative, multidisciplinary effort: scientists, clinicians, nutritionists, pharmacists, rehabilitation specialists, and patients’ families must co-design and implement treatment strategies that are individualized, targeted, and dynamically adjusted throughout recovery. To paraphrase Winston Churchill, a sentiment apt for the current state of ARDS research, this is neither the end of the fight against ARDS, nor the beginning of the end. But it is, unequivocally, the end of the beginning.

## Data Availability

Not applicable.

## Abbreviations

ARDS: Acute respiratory distress syndrome
ALI: Acute lung injury
ABG: Arterial blood gas
AEC I: Type I alveolar epithelial cell
AEC II: Type II alveolar epithelial cell
AECC: American-European Consensus Conference
AHRF: Acute hypoxemic respiratory failure
AI: Artificial intelligence
ALX: Lipoxin A4 receptor
AMs: Alveolar macrophages
Ang-2: Angiopoietin-2
ATS: American Thoracic Society
BALF: Bronchoalveolar lavage fluid
BM: Bone marrow
BMI: Body mass index
cAMP: Cyclic adenosine monophosphate
CFH: Cell-free hemoglobin
CIP: Checkpoint inhibitor pneumonitis
CMV: Cytomegalovirus
COT: Conventional oxygen therapy
CPAP: Continuous positive airway pressure
CRP: C-reactive protein
CT: Computed tomography
DAMPs and PAMPs: Damage-associated and pathogen-associated molecular patterns
DIPY: Dipyridamole
E2F2: E2F transcription factor 2
EBV: Epstein–Barr virus
ESICM: European Society of Intensive Care Medicine
EVs: Extracellular vesicles
FiO₂: Fraction of inspired oxygen
hAOs: Human airway organoids
HFNO: High-flow nasal oxygen
HMOX1: Heme oxygenase -1
HSV: Herpes simplex virus
i.v.: Intravenous
IAV: Influenza A virus
ICU: Intensive care unit
IFN-γ: Interferon-γ
IL-1: Interleukin-1
IL-10: Interleukin-10
IL-18: Interleukin-18
IL-1RA: Interleukin-1 receptor antagonist
IL-1β: Interleukin-1β
IL-4: Interleukin-4
IL-6: Interleukin-6
IL-7: Interleukin-7
IL-8: Interleukin-8
iNKT cells: Invariant natural killer T cells
iNOS: Inducible nitric oxide synthase
LPS: Lipopolysaccharide
LTs: Leukotrienes
LUS: Lung ultrasound
MALS: Macrophage activation–like syndrome
MAPK: Mitogen-activated protein kinase
MLNPs: Mannosylated lipid nanoparticles
MPO: Myeloperoxidase
MSCs: Mesenchymal stromal cells
MVECs: Murine vascular endothelial cells
NE: Neutrophil elastase
NETs: Neutrophil extracellular traps
NIV: Non-invasive ventilation
NK: Natural killer
NLRP3: NOD-, LRR- and pyrin domain-containing protein 3
NMBAs: Neuromuscular blocking agents
NRF2: Nuclear factor E2-related factor 2
PAI-1: Plasminogen activator inhibitor-1
p-AKT: Phosphorylated AKT
PaO₂: Partial pressure of arterial oxygen
PARDS: Pediatric ARDS
PBW: Predicted body weight
PDE4: Phosphodiesterase 4
PEEP: Positive end-expiratory pressure
PGs: Prostaglandins
PI3K: Phosphoinositide 3-kinase
PKA: Protein kinase A
PRRs: Pattern recognition receptors
PTEN: Phosphatase and tensin homolog deleted on chromosome 10
RACK1: Receptor for activated C kinase 1
RBC: Red blood cell
RCTs: Randomized controlled trials
recAM: Recruited alveolar macrophage
rhTM: Recombinant human thrombomodulin
RIPK3: Receptor-interacting protein kinase 3
ROS: Reactive oxygen species
RSL3: RAS-selective lethal 3
RvD1: Resolvin D1
SCCM: Society of Critical Care Medicine
SP-D: Surfactant protein-D
SpO_2_: Pulse oximetric oxygen saturation
sRAGE: Soluble receptor for advanced glycation end products
SRS: Sepsis response state
sTNFR-1: Soluble tumor necrosis factor receptor-1
TF: Tissue factor
TFPI: Tissue factor pathway inhibitor
TM: Thrombomodulin
TNF-α: Tumor necrosis factor-α
TRAF6: TNF receptor–associated factor 6
TRAMs: Tissue-resident alveolar macrophages
TXNIP: Thioredoxin-interacting protein
UC: Umbilical cord
VILI: Ventilator-induced lung injury
VT: Tidal volume
VV-ECMO: Veno-venous extracorporeal membrane oxygenation
VWF: von Willebrand factor
